# Resurrection of the *Plagiothecium longisetum* Lindb. and proposal of the new species—*P*. *angusticellum*

**DOI:** 10.1371/journal.pone.0230237

**Published:** 2020-03-11

**Authors:** Grzegorz J. Wolski, Paulina Nowicka-Krawczyk

**Affiliations:** 1 Department of Geobotany and Plant Ecology, Faculty of Biology and Environmental Protection, University of Lodz, Lodz, Poland; 2 Department of Algology and Mycology, Faculty of Biology and Environmental Protection, University of Lodz, Lodz, Poland; University of Helsinki, FINLAND

## Abstract

*Plagiothecium longisetum* was described by Lindberg in 1872, based on Maximowicz materials from Japan. In the 1970s, this species was synonymized with *P*. *nemorale*. However, a polyphasic approach applied to the investigation of the *P*. *nemorale sensu lato* showed a clear separation between the specimens of former *P*. *longisetum* and the type of *P*. *nemorale*. Morphological features and molecular analyses provide evidence that those two groups are distinct, as well as allowed to describe the new species. The results are strongly supported by the statistical analyses of morphometric features and phylogenetic analyses based on concatenated nuclear and chloroplast DNA markers. The maximum likelihood (ML) and Bayesian inference (BI) analyses of ITS, *rps4* and *rpl16* regions place both species outside the *P*. *nemorale* group. The distinctions between individual species, reflected by the morphological features—easy to observe—and the molecular data, provide a scientific foundation for the resurrection of *P*. *longisetum* Lindb. and establishment of a new species–*P*. *angusticellum sp*. *nov*.

## Introduction

*Plagiothecium* Schimp. is a pleurocarpous moss genus which belongs to the family Plagiotheciaceae M.Fleisch. The number of species in this genus is still ambiguous; estimates have ranged from 40 to even 110 species [1–3]. However, a recent revision [4] recognized 67 taxa belonging to this genus, while a further 46 names require detailed research to determine their taxonomic status. The number of recognized species has changed rapidly in recent years, which has been caused not only by an increased interest in research but also by the use of molecular methods for analysis [4–7].

One of the sections of *Plagiothecium* is *Orthophyllum* Jedl., which according to Wynns [4] includes six species. One of them is *P*. *nemorale* (Mitt.) A.Jaeger. This species was first described by Mitten [8] as *Stereodon nemoralis*, and its current synonymy is: *P*. *longisetum* Lindb. [9], *P*. *sylvaticum* var. *nemorale* (Mitt.) Paris [10], *P*. *sylvaticum* var. *rhynchostegioides* Cardot, *P*. *sylvaticum* var. *latifolium* Cardot [11], *P*. *neglectum* Mönk. [12], *P*. *saxicola* Sak. [13], *P*. *longisetum* var. *brevinerve* Iisiba [14].

For the last 50 years [3, 15–18], scientists have indicated that *P*. *nemorale* is a very variable species; however, it has never been the subject of a detailed study. The research of Wolski [19–21] and Wolski et al. [22] on intraspecific variability of *P*. *nemorale sensu lato* not only pointed to the heterogeneous nature of this species but also allowed the distinction of two groups of specimens within this taxon. The groups differ in both qualitative and quantitative characteristics of the gametophyte. The features that have the greatest value for the discrimination of these groups are the length and width of the leaf cells [19].

The dimensions of the cells located in the middle part of the leaf are the most important taxonomic features for the species belonging to sect. *Orthophyllum* and for all of *Plagiothecium* [3, 15–18, 23–24]. In addition, Wolski [19–21] has pointed out that the dimensions and shape of cells from other leaf zones (the apex and base) can also play a diagnostic role.

Detailed analyses of herbarium specimens, including available types, among others: *S*. *nemoralis*, *P*. *saxicola*, *P*. *longisetum*, as well as protologues of synonyms of *P*. *nemorale* [9–14], has indicated that these specimens differ in both qualitative and quantitative characteristics, including leaf cell dimensions. The existing differences correspond to the differences indicated by Wolski [19, 21], and the two groups distinguished by this author can be assigned to two separate species: *P*. *nemorale sensu stricto* and *P*. *longisetum*. Thus, these studies indicated that *P*. *nemorale* is a complex.

*Plagiothecium longisetum* was described by Lindberg [9] based on materials collected in 1863 by Maximowicz from the island of Kiusiu (Japan). He characterized this species, among others, as a species whose turfs are white-greenish or yellow-green “lurido- vel fulvo-viridulos,” while the leaves in dry conditions are usually gently shrunken and corrugated “vulgo leniter secunda, sicca leniter torta et undulata,” slightly asymmetrical and ovate “paullo asymmetrica, ovata,” with a smooth (…) margin “margine (…) te ubique integerrimo.” Whereas cells are very loose and wide “cellulis duplo latioribus, laxissimae, magnae”.

Subsequent to its description, *P*. *longisetum* not only appeared in studies documenting bryophytes from various parts of the world [25–26] but was listed in the most important bryological checklists of that time [10, 27]. This situation changed after a taxonomic revision of this genus, when Zennoske Iwatsuki proposed the synonymization of *P*. *longisetum* with *P*. *nemorale* [16]. That synonymy has remained unchallenged [28].

The aim of this article is to demonstrate that *P*. *longisetum* is a well-defined species and to restore it as a separate one, independent from *P*. *nemorale*. In addition, the purpose of the article is to describe a new species of *P*. *angusticellum* and to indicate differences between these closely related taxa.

## Materials and methods

### Taxonomic analyses

During the research, 3000 specimens of *P*. *nemorale sensu lato* from throughout its range in Eurasia were revised. The tested specimens came from the following 34 herbaria: AAU, BG, BM, BRA, BRNU, C, CP, E, GB, H, IBL, KRAM B, LBL, LOD, MANCH, NTNU, NY, OXF, PL, POZG-B, PR, PRC, S, SLO, SOSN, TAA, TALL, TAM, TRH, TROM, TU, TUB, UME, UPS. The following available types were also analyzed: *S*. *nemoralis* (NY *913349*), *P*. *saxicola* (PC*132573*), *P*. *longisetum* (PC*132572*, H-SOL*1563011*), as well as the protologues of synonyms of *P*. *nemorale* [9–14].

### Statistical analyses

A representative group of 240 specimens was selected for statistical analysis from the entire geographical range of the studied taxon. The statistical analysis included the two most important specimens for these studies, the types (*S*. *nemoralis* NY *913349*, and *P*. *longisetum* PC*132572*) and all specimens used for molecular analysis (Wolski1, Wolski5, Wolski12, Wolski14–15, Wolski17, Wolski19, Wolski22–26, Wolski28–29). The list of all examined specimens (S1 Text) and raw morphological data (S1 Raw Data) are available as supplementary materials.

Wolski’s previous research [19] indicated that the best features to capture intraspecific variability of *P*. *nemorale sensu lato* are associated with leaf cells; therefore, only these features were considered in the present investigation (Table 1). Among the selected specimens, one stem was chosen from uniform turf. The leaves were torn off from the central part of the stem, and for each leaf, the length and width of five randomly selected cells were measured. The cells were measured: in the upper, middle, and lower part of the leaf. The method of measurement and location of the characteristics examined on the leaf was described in detail in Wolski’s [19] article.

**Table 1:** The measured features and their symbols.

Symbols	Characteristic
**LC1**	The length of the cells from the top part of the leaf.
**WC1**	The width of the cells from the top part of the leaf.
**LC2**	The length of the cells from the middle part of the leaf.
**WC2**	The width of the cells from the middle part of the leaf.
**LC3**	The length of the cells from the lower part of the leaf.
**WC3**	The width of the cells from the lower of the leaf.

Quantitative variables were characterized by providing basic descriptive statistics: the number of observations, mean, median, minimum and maximum, first (Q1) and third (Q3) quartiles, and standard deviation (SD). Variable distributions were presented in the form of density curves obtained as a result of nuclear estimation (Gauss function, smoothing SROT method) [29]. The normality of the distributions was tested by the Shapiro-Wilk test. To determine the inclusion of objects in groups (clusters of points corresponding to objects), principal components analysis (PCA), grouping by the k-means method and hierarchical cluster analysis (HCA)–Ward’s method and the Euclidean distance–were used. Student’s t-test was used to determine the statistical significance of differences between the groups (with the Cochran–Cox correction). In the above test, the results where the significance level was lower than 0.05 were considered statistically significant results. As a measure of the effect, Cohen’s d was adopted. Calculations were made using statistical packages STATISTICA v. 13—TIBCO Software Inc. (in Poland StatSoft Polska) and PQSTAT v.1.6.8. (PQStat Software).

### DNA isolation, amplification and sequencing

Green leafy stems of mosses collected by G. J. Wolski in 2017 and 2018 were cut from dried material under the inverted microscope NIKON Eclipse Ts2 (Precoptic Co., Warsaw, Poland) to avoid contamination by other organisms and to exclude any debris. Approximately 20 mg of dry tissue from each specimen in duplicates was placed in a 1.5 ml Eppendorf Safe-Lock tube and frozen (-20°C) for homogenization. Tissue homogenization was performed using a hand-held stainless steel homogenizer (Schlüter Biologie, Eutin, Germany) until a more or less homogenous dry powder was obtained. Total DNA was extracted using the GeneMATRIX Plant & Fungi DNA Purification Kit (Eurx, Gdansk, Poland) following the manufacturer’s protocol. DNA extracts were quantified with a BioDrop DUO Spectrophotometer (BioDrop Ltd, Cambridge, UK). From the duplicates, the sample with higher quality DNA (1.7–1.9 OD_260_/OD_280_) was selected for further analysis.

The molecular research was based on nuclear and chloroplast DNA markers: ITS (from the 3' end of the hypervariable nuclear spacer ITS1, through the 5.8S gDNA, to the 5`end of the ITS2 spacer); *rpl16* cpDNA gene encoding ribosomal protein L16; and *rps4* cpDNA gene encoding ribosomal protein S4. Markers were selected based on Wynns et al. [5] and Ignatova et al. [7], which focus mainly on the genus *Plagiothecium*.

For each sample, all markers were amplified by PCR in a few replicates to obtain high quality amplicons for sequencing. PCR was performed using primers and reaction conditions as described in Table 2, with a 50 μl reaction volume with 25 μl of Color Taq PCR Master Mix (2×) (Eurx, Gdansk, Poland).

**Table 2:** Primers used for amplification and sequencing with PCR reaction conditions.

Marker	Primer	F/R	Concentr. [pmol μl^−1^]	Sequence reference	Reaction conditions
**ITS**	m-18-s	F	7.5	Spagnuolo et al. [30]	95°C(3m); 35×[95°C(1m)/52°C(1m)/72°C(1.5m)]; 72°C(7m)
	ITS1	F	7.5	Wynns et al. [5]	
	LS4-R	R	7.5	Shaw [31]	
**rpl16**	F71	F	5	Jordan et al. [32]	94°C(1m); 35×[95°C(0.5m)/56°C(1m)/68°C(1.5m)]; 68°C(4m)
	rpl16R	R	5	Olsson et al. [33]	
**rps4**	trnS	F	5	Wynns & Lange [34]	94°C(3m); 35×[94°C(0.5m)/50°C(0.5m)/72°C(1m)]; 72°C(5m)
	rps5'	R	5	Wynns & Lange [34]	

PCR products were visualized on an agarose gel (1.5%, 90V, 40 minutes) stained with GelRED^™^ fluorescent dye (Biotum, Fremont, CA, USA) and two replicates of each marker per sample were chosen for sequencing. Amplicons after PCR reaction were cleaned using Syngen Gel/PCR Mini Kit (Syngen Biotech, Wroclaw, Poland) according to the manufacturer’s protocol. Samples were sequenced with Sanger sequencing using primers from amplification by SEQme s.r.o. company (Dobris, Czech Republic). The obtained sequences were assembled in Geneious 11.1.5 (Biomatters Aps, Aarhus, Denmark) (http://www.geneious.com) and the genetic distance of ITS-*rps4*-*rpl16* matrix between studied taxa was calculated using MEGA X software [35]. The sequences were submitted to the NCBI GenBank database (www.ncbi.nlm.nih.gov) under the accession numbers MN077500–MN077513 for ITS and MN311135–MN311162 for *rpl16* and *rps4*.

### Phylogenetic analyses

Two phylogenetic analyses were performed. The first one was based on the ITS sequences of *P*. *longisetum* specimens, other specimens of *Plagiothecium*, and similar hypnalean mosses. The second one included, in addition to ITS, *rps4* and *rpl16* chloroplast markers of *P*. *longisetum* specimens and other in the *Plagiothecium* group. Voucher information for the specimens included in this study, with corresponding GenBank accession numbers, are presented in ITS phylogenetic tree figure and Table 3 (*rps4* and *rpl16*). Sequences were aligned using the MAFFT v. 7 web server [36] (http://mafft.cbrc.jp/alignment/server/) where the auto strategy was applied, the scoring matrix of 200PAM with Gap opening penalty of 1.53, UniREf50 for Maft-homologs and Plot and alignment with threshold of 39 score were set. The obtained alignments were checked for poorly and ambiguously aligned regions and small corrections were made by eye. Phylogenetic calculations were performed using maximum likelihood analysis (ML) in the IQ-TREE web server [37] (http://iqtree.cibiv.univie.ac.at/) with the ultrafast bootstrap (UFBoot) pseudolikelyhood algoritm [38] and 1000 replicates; and Bayesian inference (BI) in MrBayes 3.2.2 [39], where two parallel Markov chain Monte Carlo (MCMC) runs for one million generations each, with trees sampled every 100 generations were performed. The average standard deviation of split frequencies in both cases remained below 0.01 for the last 1000 generations and posterior probabilities were estimated from the 50% majority-rule consensus tree after elimination of the first 25% of samples as burn-in. The evolutionary models were calculated using PartitionFinder 2 software [40], chosen according to the Akaike Information Criterion for the ITS set and ITS-*rps4*-*rpl16* matrix (S1 Table) and the phylogenetic trees were constructed using a set of partitions [41]. The alignments and tree files were submitted to figshare online database (https://doi.org/10.6084/m9.figshare.11882217.v1).

**Table 3:** Voucher information and accession numbers for the specimens included in the phylogenetic analysis of chloroplast markers.

Taxon	Collection	Locality	rpl16	rps4
***Plagiothecium piliferum***	J. Shevock 26205	WA, USA	KF882340	KF882365
***Plagiothecium nemorale***	J.T. Wynns 2684	Germany	KF882337	KF882362
***Plagiothecium cavifolium***	J.T. Wynns 2960	Germany	KF882326	KF882351
***Plagiothecium cavifolium***	J.T. Wynns 1885	Denmark	KF882325	KF882350
***Plagiothecium curvifolium***	J.T. Wynns 1939	Denmark	KF882327	KF882352
***Plagiothecium curvifolium***	G. Rothero s.n.	Germany	KF882328	KF882353
***Plagiothecium denticulatum***	J.T. Wynns 2081	Denmark	KF882329	KF882354
***Plagiothecium denticulatum* var *obtusifolium***	J.T. Wynns 2842	Germany	KF882330	KF882355
***Plagiothecium draytonii***	W.J. Hoe 3557	HI, USA	KF882331	KF882356
***Plagiothecium euryphyllum***	D.G. Long 36218	China	KF882332	KF882357
***Plagiothecium handelii***	D.G. Long 34930	China	KF882333	KF882358
***Plagiothecium laetum***	J.T. Wynns 2907	Germany	KF882334	KF882359
***Plagiothecium latebricola***	I. Goldberg s.n.	Denmark	KF882335	KF882360
***Plagiothecium neckeroideum***	J. Shevock 26916	China	KF882336	KF882361
***Plagiothecium nemorale***	B. Mishler 3835	Iran	KF882338	KF882363
***Plagiothecium nemorale***	J.T. Wynns 3044	Germany	KF882339	KF882364
***Plagiothecium platyphyllum***	J. Lewinsky s.n.	Finland	KF882341	KF882366
***Plagiothecium ruthei***	J.T. Wynns 1997	Denmark	KF882342	KF882367
***Plagiothecium undulatum***	J.T. Wynns 2050	Denmark	KF882344	KF882370
***Pseudotaxiphyllum elegans***	J.T. Wynns 3061	Germany	KF882346	KF882371
***Plagiothecium longisetum***	G. J. Wolski 14 specimens	Poland	MN311135-MN311148	MN311149-MN311162

### Nomenclature

The electronic version of this article in Portable Document Format (PDF) in a work with an ISSN will represent a published work according to the International Code of Nomenclature for algae, fungi, and plants, and hence the new names contained in the electronic publication of a PLOS article are effectively published under that Code from the electronic edition alone; there is no longer any need to provide printed copies.

New names contained in this work have been submitted to IPNI, from where they will be made available to the Global Names Index. The IPNI LSIDs can be resolved and the associated information viewed through any standard web browser by appending the LSID contained in this publication to the prefix http://ipni.org/. The online version of this work is archived and available from the following digital repositories: (PubMed Central, LOCKSS).

## Results

Type specimens of *S*. *nemoralis* (NY *913349*), *P*. *saxicola* (PC*132573*) and *P*. *longisetum* (PC*132572*, H-SOL *1563011*) differed in a number of qualitative and quantitative features. The most important of these were: the shape and symmetry of the leaf, the shape and serration of the leaf apex, as well as the shape, length and width of leaf cells. These differences are noticeable in specimens from the entire geographical range of *P*. *nemorale sensu lato*.

In the studied specimens of *P*. *nemorale sensu lato*, the range of variability of cell length (LC1, LC2, LC3) is variable, and widest for cells located in the lower (LC3) and the top part of the leaf (LC1) (S2 Table). An analysis of the distributions of the studied variables indicates that they are multimodal. Among the examined specimens, two groups can be identified that are significantly different in terms of cell length (S1 Fig).

**Fig 1:**
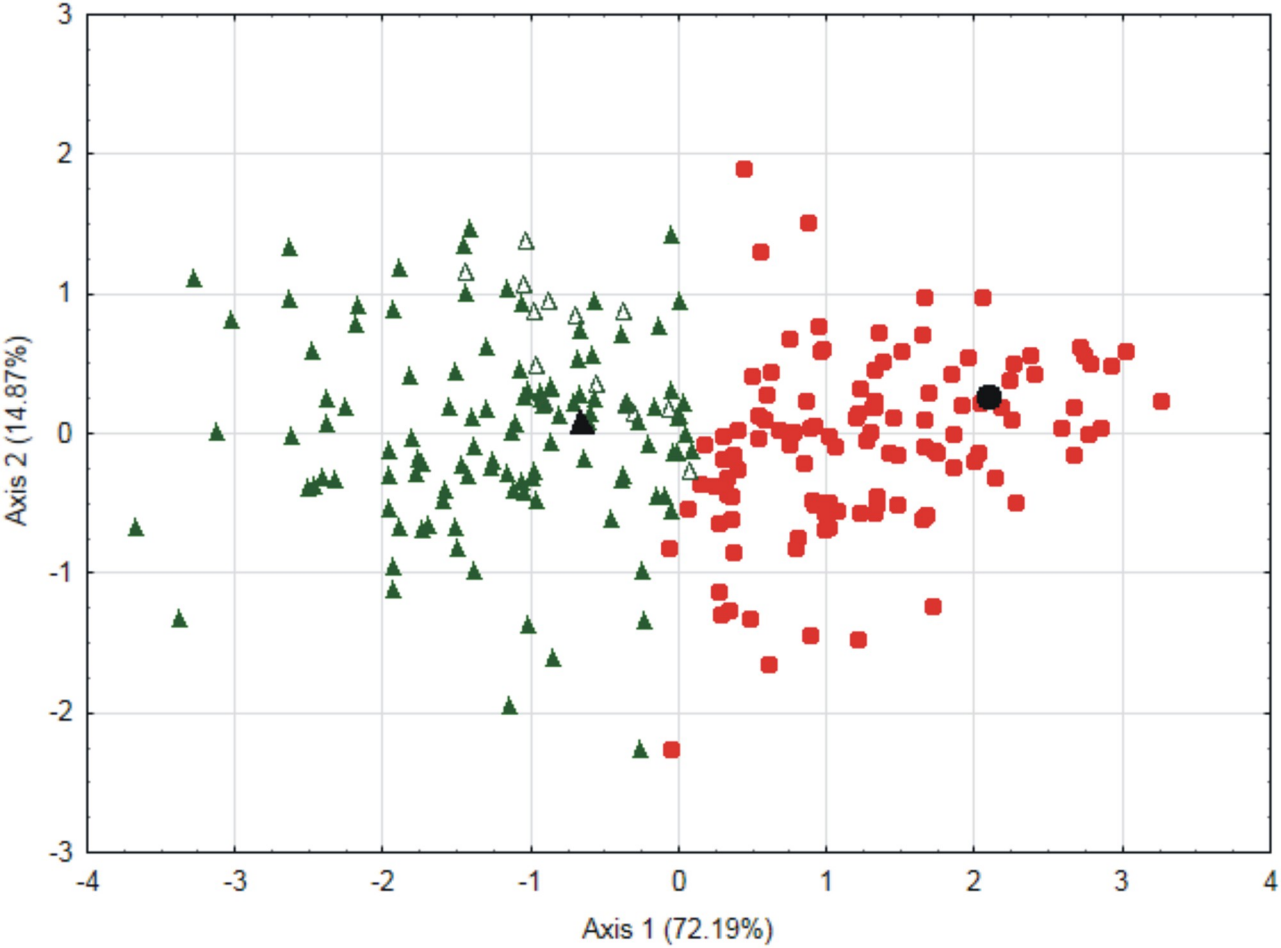
PCA of the leaf cell width and length measurements of all tested *P*. *nemorale sensu lato* specimens. Green triangles–group of specimens of *P*. *longisetum*, white triangles–molecularly examined specimens of *P*. *longisetum* (Wolski1, Wolski5, Wolski12, Wolski14-15, Wolski17, Wolski19, Wolski22-26, Wolski28-29), black triangle–*P*. *longisetum* type, red circles–group of specimens of *P*. *nemorale sensu stricto*, black circle–*P*. *nemorale* type.

Principal components analysis (PCA) in which cell length were taken into account was used to organize specimens. The results indicate that the two axes explained 87.06% of variability (PC1 explains 72.19%, PC2 explains 14.87% of variability). Grouping using the k-means method gave a division into two groups. Specimens from one group can be assigned to *P*. *nemorale sensu stricto*, while those from the other group are *P*. *longisetum* (Fig 1).

The biggest difference between specimens of *P*. *nemorale sensu stricto* and *P*. *longisetum* is the length of their cells. Specimens of *P*. *nemorale* have short cells ($\bar{\text{x}}$: LC1 78.1, LC2 96.7, LC3 121.6) while specimens of *P*. *longisetum* have long cells ($\bar{\text{x}}$: LC1 104.6, LC2 128.9, LC3 154.7) (S3 Table).

*Plagiothecium longisetum* Lindb., Acta Soc. Sci. Fenn. 10: 232 (1875). Type: Japan, ad Nikosan ins. Kiusiu. fertile. 16 Junii 1863. *S*.*O*. *Lindberg*.

Reference sequence–specimen Wolski19: MN077506 (ITS), MN311155 (*rps4*), MN311141 (*rpl16*).

Fig 2

**Fig 2:**
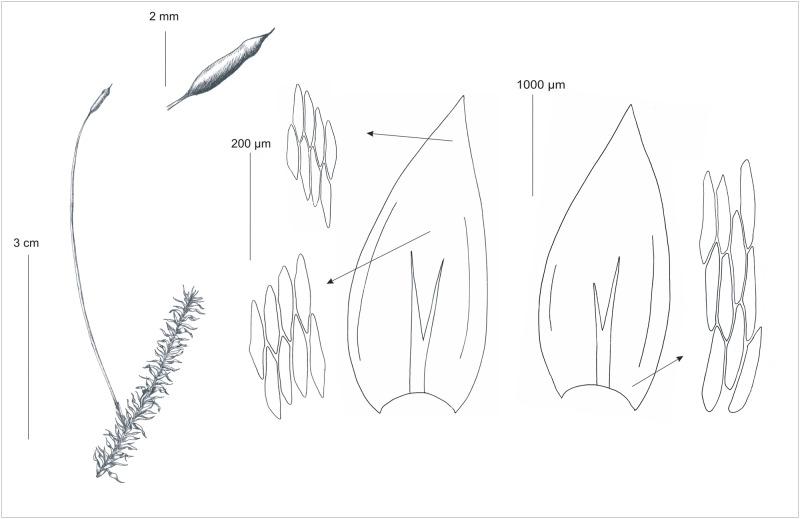
Gametophyte and sporophyte as well as stem leaves and cells from individual zones of the *P*. *longisetum*. Drawn from the type material (PC*132572*, H-SOL *1563011*) G. J. Wolski & A. Cienkowska.

Plants medium-sized to large, green to yellowish, without metallic luster. Stems 2–3 cm long, more or less complanate-foliate, in cross-section rounded, with a diameter of 374.2–641.9 ($\bar{\text{x}}$ 541.6) μm, central strand developed, epidermal cells 10.7–28.5 ($\bar{\text{x}}$ 17.6) × 11.9–34.3 ($\bar{\text{x}}$ 24.5) μm, parenchyma thin-walled, 15.6–61.1 ($\bar{\text{x}}$ 41.8) × 24.9–41.6 ($\bar{\text{x}}$ 41.6) μm; in dry conditions leaves shrunken, leaves concave, generally strongly asymmetrical, ovate to lanceolate, those from the middle of the stem 3–4 ($\bar{\text{x}}$ 3.5) mm long, and the width measured at the widest point 1.6–2 ($\bar{\text{x}}$ 1.7) mm; the apex acute to acuminate; margins not denticulate near the apex; costae 2, strong, extending to ½ of the leaf length or even more, reaching 1.1–2.4 ($\bar{\text{x}}$ 1.5) mm; laminal cells elongate-hexagonal, in irregular transverse rows, the length and width variable depending on location: 68.5–158.1 ($\bar{\text{x}}$ 104.9) × 17–32.3 ($\bar{\text{x}}$ 24.7) μm at the apex, 94.6–150.3 ($\bar{\text{x}}$ 129.9) × 17–34.1 ($\bar{\text{x}}$ 25.9) μm at the midleaf, and 96.1–223.1 ($\bar{\text{x}}$ 159.1) × 19.9–40.2 ($\bar{\text{x}}$ 29.3) μm at the lower part of the leaf, due to the fact that cells are long and wide, the areolation is very lax; decurrencies of 3 rows of rectangular cells, best seen while still attached to the stem. 70.1–149.6 ($\bar{\text{x}}$ 102.8) × 17.1–34 ($\bar{\text{x}}$ 25.8) μm. The seta is smooth, straight, and orange-reddish, 4.5–5.5 cm long. Capsule is inclined to horizontal, and has a cylindrical shape, even when dry are smooth and dark brown, 2.5–2.7 × 0.5–0.7 mm long (immature capsules without the operculum). Exothecial cells are thin-walled, and rectangular, less quadratic, 43.7–98.6 ($\bar{\text{x}}$ 74.2) × 24.3–50.4 ($\bar{\text{x}}$ 41) μm. The operculum is rostrate in shape and reaches 1.3–1.5 mm long. The annulus is composed of 2–3 rows of cells, 25–50 × 12.5–17.5 μm. The double peristome is well developed, the exostome teeth are lanceolate, narrowly triangular, and bright orange, 584.2–606 × 107.2–121.8 μm. The outer surface of the exostome teeth is cross-striolate and at back they are trabeculate. The endostome is yellowish and has a triangular prism shape. The segments are almost as long as the exostome teeth, 612–644 μm. Spores are spherical, their diameter ranges from 10–12.5 μm.

**Fig 3:**
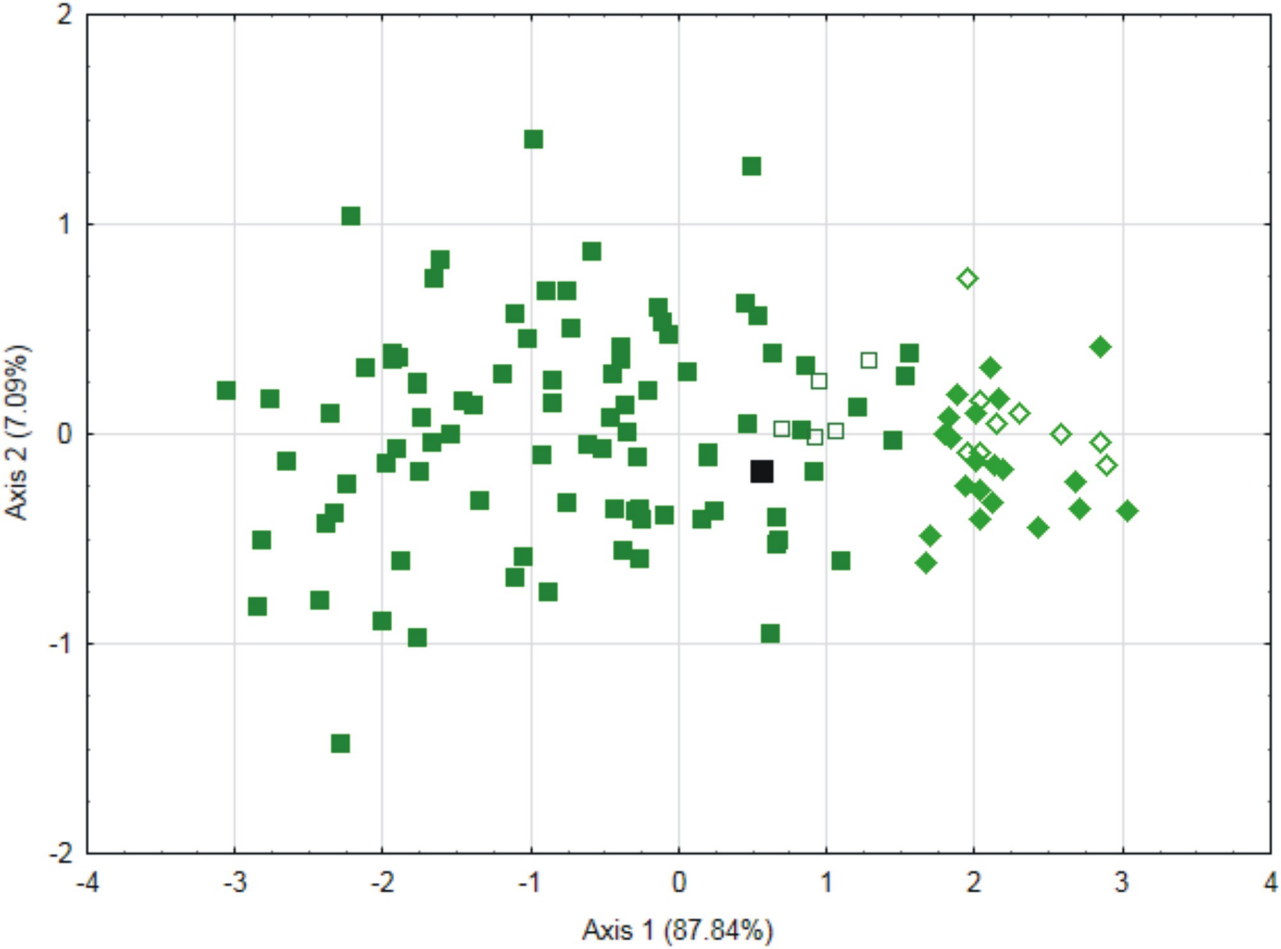
PCA of the leaf cell width and length measurements of *P*. *longisetum* and *P*. *angusticellum* specimens. Green squares–*P*. *longisetum*, white squares–molecularly examined specimens of *P*. *longisetum* (Wolski12, Wolski14, Wolski15, Wolski17, Wolski19), black square–*P*. *longisetum* type, green rhombus–*P*. *angusticellum*, white rhombus–molecularly examined specimens of *P*. *angusticellum* (Wolski1, Wolski5, Wolski22-23, Wolski24-26, Wolski28-29).

The analysis of variable distributions for the species indicates that the distribution of *P*. *nemorale sensu stricto* is quite homogeneous but bimodal for *P*. *longisetum*, with two clusters forming. The heterogeneity of this taxon is also supported by molecular analysis. Two clustering within *P*. *longisetum* arise because of the width of the leaf cells (WC1, WC2, WC3) (S2 Fig). Due to this fact, in the next PCA analysis, only individuals belonging to the *P*. *longisetum* group were considered along with features associated with cell width (WC1, WC2 and WC3). The two major axes explain 94.93% of variability (PC1 explains 87.84% while PC2 explains 7.09% of variability. A division into two groups was obtained by using the HCA method. The first group includes specimens with long and wide cells (*P*. *longisetum*), while the other contains specimens with long and narrow cells–*P*. *angusticellum sp*. *nov*. (Figs 3 and 5).

Specimens of *P*. *longisetum* differ from *P*. *angusticellum* in terms of cell length (*P*. *longisetum $\bar{\text{x}}$*: LC1 104.9, LC2 129.9, LC3 159.1; *P*. *angusticellum $\bar{\text{x}}$*: LC1 103.7, LC2 125.9, LC3 140.8). However, the biggest difference relates to their width. Specimens of *P*. *longisetum* are characterized by wide cells ($\bar{\text{x}}$: WC1 24.7, WC2 25.9, WC3 29.3), versus narrow cells in *P*. *angusticellum* ($\bar{\text{x}}$: WC1 16.7, WC2 17.4, WC3 20.3) (S4 Table). In addition, the Student’s t-test indicates that for the studied groups there are significant differences between the values of variables LC2, LC3, WC1, WC2 and WC3. The largest effect is primarily for the features associated with the cell width of these specimens (S5 Table).

**Fig 4:**
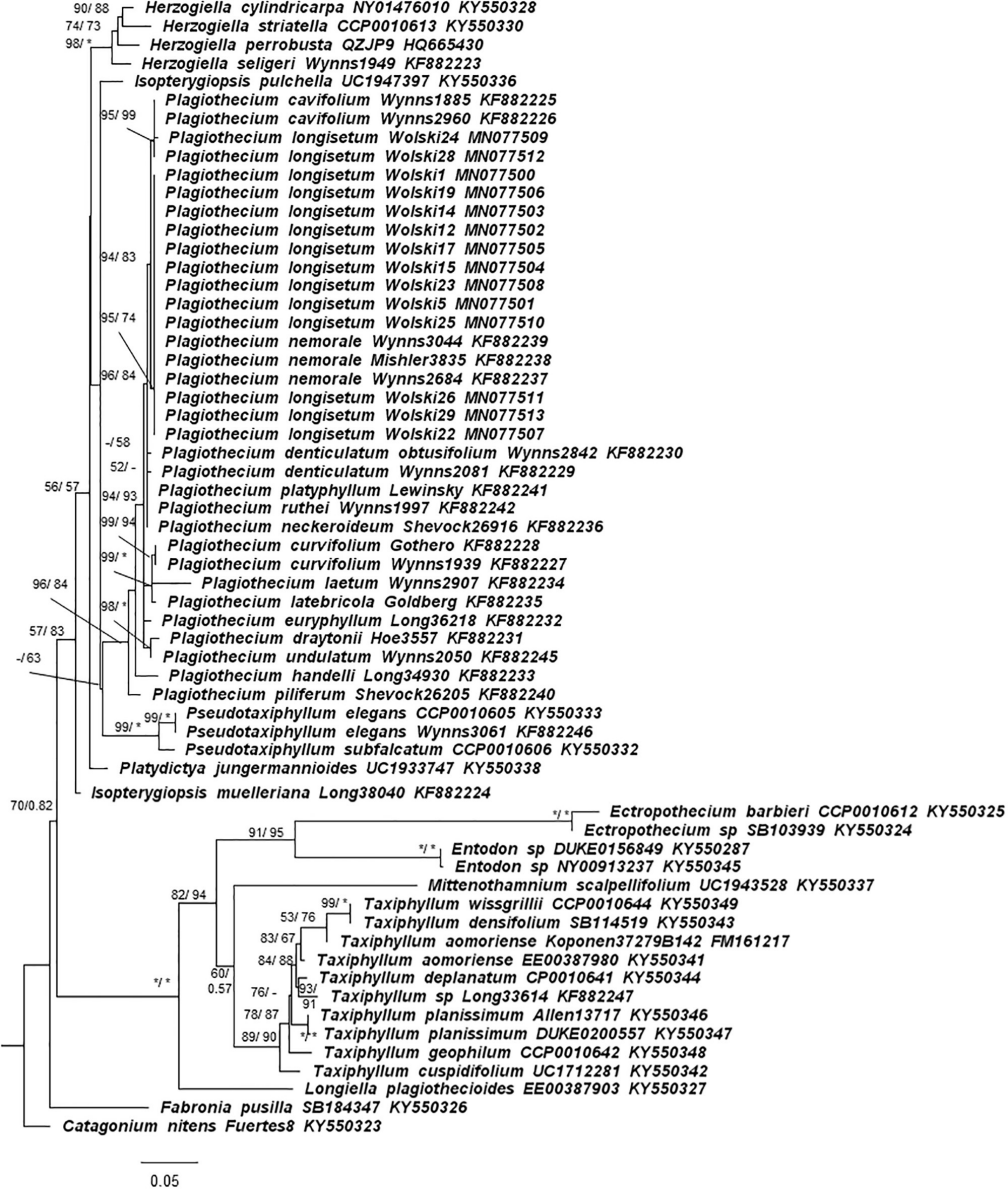
Phylogenetic tree based on ITS sequences (total 710 bp) showing the position of *P*. *longisetum* specimens among other *Plagiothecium* and similar hypnalean mosses. Numbers on branches indicate bootstrap values from ML followed by posterior probabilities from BI analysis. Asterisk (*) indicates 100 (ML) and 1.00 (BI), while minus (-) indicates values below 50 (ML, BI). The topology of the tree was based on ML analysis.

**Fig 5:**
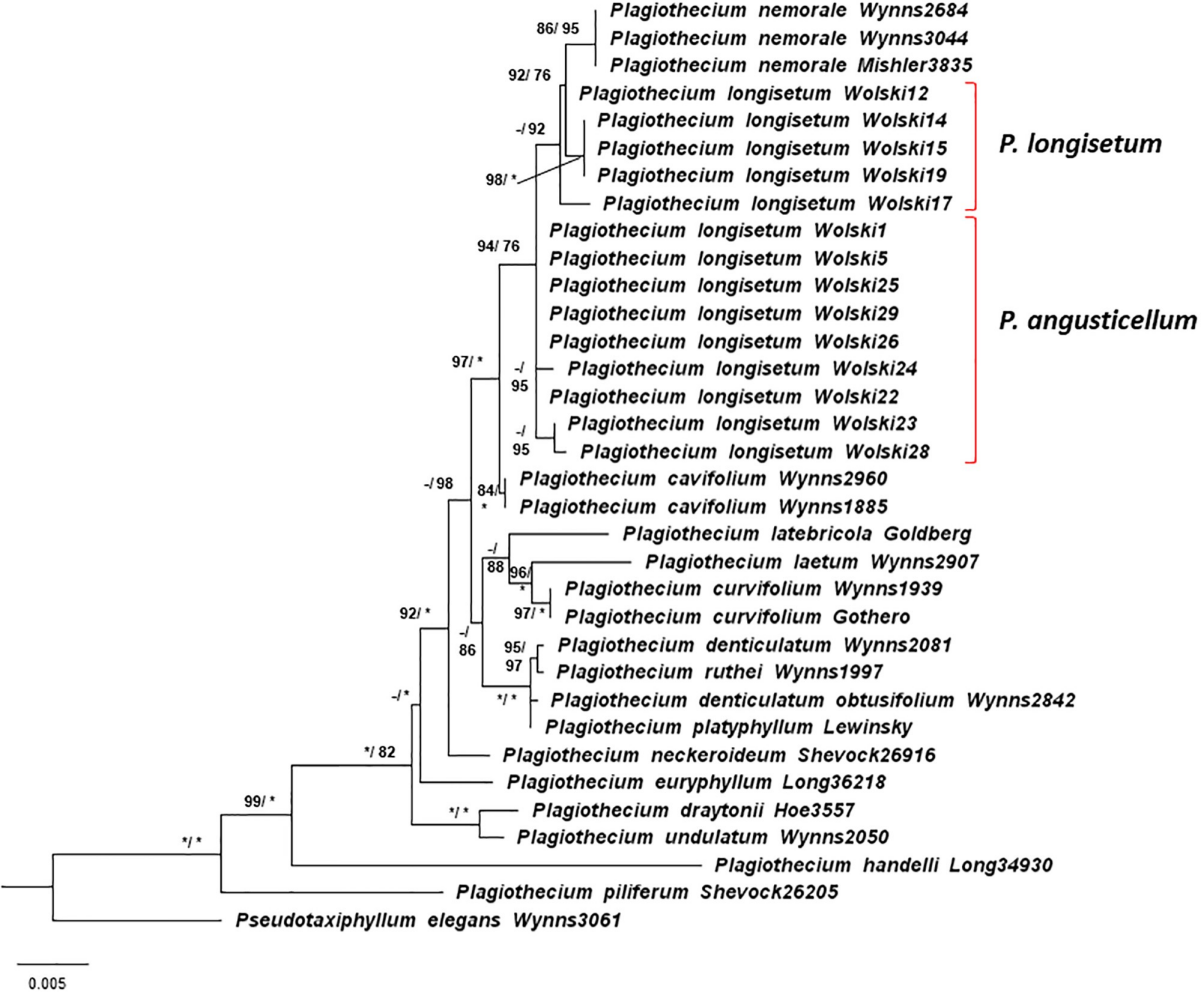
Phylogenetic tree of *Plagiothecium* group with *Pseudotaxiphyllum* as the outgroup taxa based on concatenated nuclear (ITS) and chloroplast (*rps4* and *rpl16*) DNA markers (total 2068 bp). **The tree shows the position of *P*. *longisetum* specimens among the *Plagiothecium* group.** Numbers on branches indicate bootstrap values from ML followed by posterior probabilities from BI analysis. Asterisk (*) indicates 100 (ML. MP) and 1.00 (BI), while minus (-) indicates values below 80 (ML) and 75 (BI). The topology of the tree was based on ML analysis.

Phylogenetic analyses based on the ITS region placed most of the sequences of ‘*P*. *longisetum*’ Wolski specimens in a clade with *P*. *nemorale* (Fig 4). Only two specimens (Wolski24 and Wolski28) were grouped outside the clade, together with other specimens of *Plagiothecium*. However, the concatenated analysis of ITS and chloroplast DNA markers *rps4* and *rpl16* placed Wolski specimens outside the *P*. *nemorale* clade. Moreover, the analysis revealed that 5 specimens described as Wolski 12, 14, 15, 19 and 17 –*P*. *longisetum*–are more closely related to *P*. *nemorale*, than others–*P*. *angusticellum* (Fig 5). Genetic distance between *P*. *nemorale* and Wolski specimens based on the concatenated matrix was between 0.0026 and 0.0078 (S6 Table). Lower distance was recorded for *P*. *longisetum*–up to 0.0047, while higher for the *P*. *angusticellum* (Wolski 1, 5, 22, 23, 24, 25, 26, 28, 29)–from 0.0052. The separateness of both groups in concatenated matrix is most strongly confirmed by the *rpl16* marker (S7–S9 Tables).

**Fig 6:**
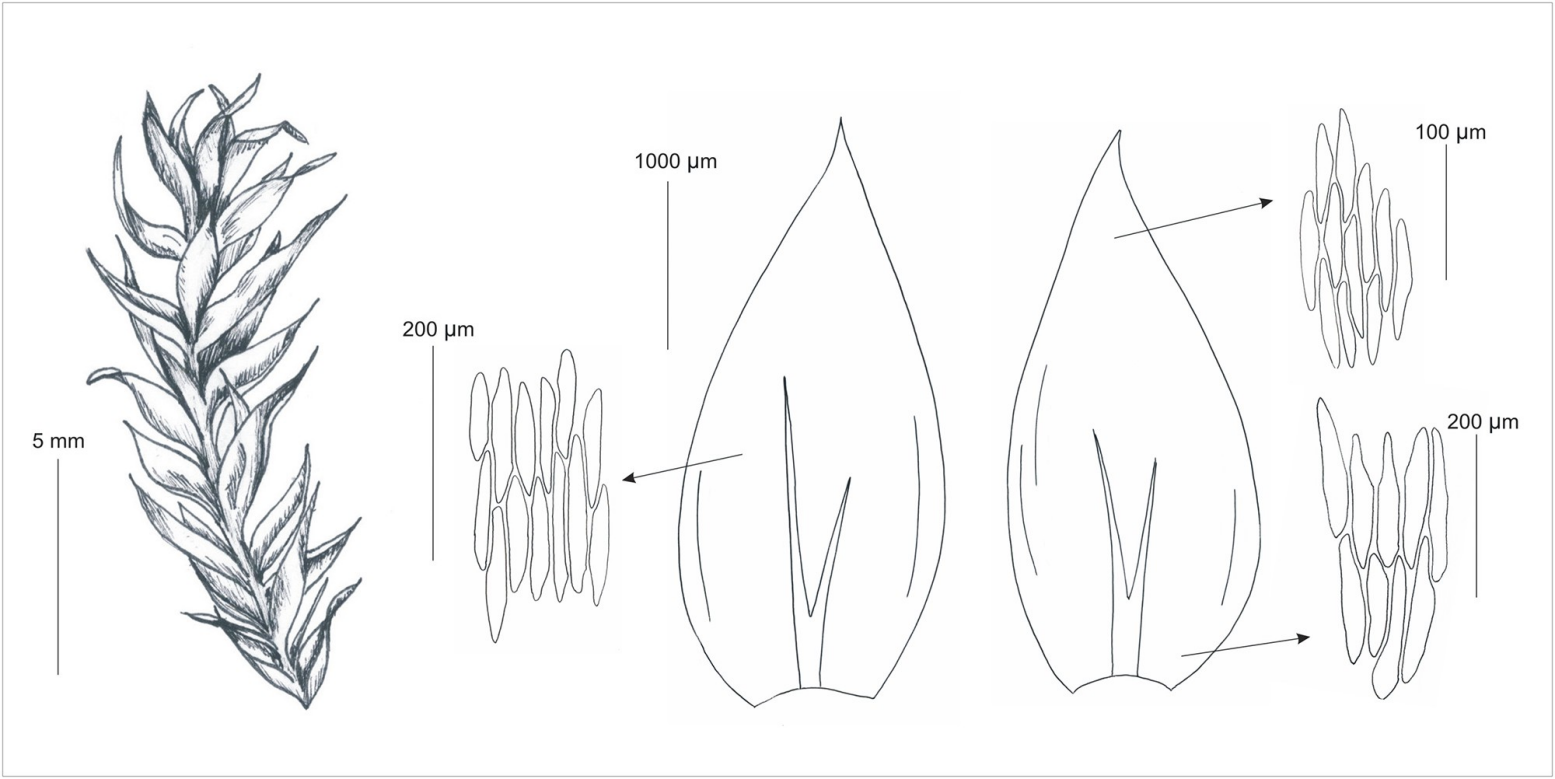
Gametophyte, stem leaves and cells from individual leaf zones of the *P*. *angusticellum*. Drawn from the holotype (LOD 14927) G. J. Wolski & A. Cienkowska.

Based on the phylogenetic analyses (Fig 5) and morphological distinctions (Figs 2, 3 and 6) from the *P*. *nemorale sensu lato* we propose establishment of a new species–*Plagiothecium angusticellum sp*. *nov*.

*Plagiothecium angusticellum* G. J. Wolski & P. Nowicka-Krawczyk *sp*. *nov*.

Type: Poland. Łódzkie Voivodeship. Grądy nad Moszczenicą reserve, 51°55′N, 19°29′E, at the base of *Carpinus betulus* in *Fraxino-Alnetum* forest, 11 Dec 2017, *G*. *J*. *Wolski* (Holotype LOD *14927*, Isotype LOD *14937*).

Reference sequence—specimen Wolski22: MN077507 (ITS), MN311156 (*rps4*), MN311142 (*rpl16*).

Fig 6

Etymology: Angustus- [Lat.] narrow; -cellus [Lat.] cell. The presented species is named in reference to the most distinctive feature easily noticeable–its narrow cells.

Plants up to 4 cm long, without metallic luster. Leaves ovate to lanceolate, concave, generally asymmetrical, in dry conditions not shrunken. The apex acuminate, often gently curved; margins not denticulate. Laminal cells narrowly elongate-hexagonal, gently asymmetric, in irregular transverse rows, cells 113–143.3 ($\bar{\text{x}}$ 125.9) × 15.1–19.3 ($\bar{\text{x}}$ 17.4) μm at the mid-leaf, cell areolation dense.

Plants medium-sized to large, dark green, dull, without metallic luster. Stems 2–4 cm long, more or less complanate-foliate, in cross-section rounded, with a diameter of 332.1–446.7 ($\bar{\text{x}}$ 398.7) μm, the central strand developed, epidermal cells 9.1–21.1 ($\bar{\text{x}}$ 14.1) × 14.6–25.4 ($\bar{\text{x}}$ 20.6) μm, the parenchyma thin-walled, 12.2–51.3 ($\bar{\text{x}}$ 33.2) × 17.5–55 ($\bar{\text{x}}$ 33.7) μm; in dry conditions leaves not shrunken, leaves concave, generally asymmetrical, ovate to lanceolate, those from the middle of the stem 3.1–3.4 ($\bar{\text{x}}$ 3.3) mm long, and the width measured at the widest point 1.3–1.5 ($\bar{\text{x}}$ 1.4) mm; the apex acuminate, often gently curved; margins not denticulate near the apex; costae 2, extending to ½ of the leaf length, reaching 0.7–1.4 ($\bar{\text{x}}$ 1.1) mm; laminal cells narrowly elongate-hexagonal, gently asymmetric, in irregular transverse rows, the length and width variable depending on location: 81.7–120.4 ($\bar{\text{x}}$ 103.7) × 13.2–19.5 ($\bar{\text{x}}$ 16.7) μm at the apex, 113–143.3 ($\bar{\text{x}}$ 125.9) × 15.1–19.3 ($\bar{\text{x}}$ 17.4) μm at the midleaf, and 123.1–172 ($\bar{\text{x}}$ 140.8) × 16.4–24.6 ($\bar{\text{x}}$ 20.3) μm at the lower part of the leaf, due to the fact that cells are long and quite narrow, cell areolation dense; decurrencies of 3 rows of rectangular to quadrate cells, best seen while still attached to the stem, 43.1–105.5 ($\bar{\text{x}}$ 70.8) × 17.8–34.5 ($\bar{\text{x}}$ 27.8) μm. Sporophytes not seen.

In terms of shape, ovate to lanceolate leaves of *Plagiothecium angusticellum* are quite similar to *P*. *longisetum* leaves [9], however, they are distinctly different from very ovate leaves of *P*. *nemorale* [3, 16, 18]. The asymmetry of the leaves of the new species also refers to *P*. *longisetum*, and distinguishes this species from the symmetrical leaves of other species forming the *Orthophyllum* section, for example: *P*. *nemorale*, *P*. *cavifolium* (Brid.) Z.Iwats., *P*. *succulentum* Wilson (Lindb.) [3, 16, 18, 20]. Similarly, the shape of the leaf apex clearly distinguishes *P*. *angusticellum* among those previously mentioned. Acuminate, gently curved, and not denticulate apex of the new species is a unique combination. Comparing these characteristics, *P*. *longisetum* has a straight, acute to acuminate and not denticulate apex [9], while, *P*. *nemorale* has a straight, acute, apiculate and denticulate apex [3, 16, 18]. Long cells (113–143.3 μm; $\bar{\text{x}}$ 125.9) (S4 Table) of new species make it similar to *P*. *longisetum*, and distinguish this taxon from *P*. *nemorale* (which in the middle part of the leaf has cells up to 100 μm). Additionally, *P*. *angusticellum* is distinguished from the other species by the width of cells. In the central part of the leaf, they are narrow 15.1–19.3 μm ($\bar{\text{x}}$ 17.4), while in the closest related species they reach from 12.1–32.1 μm ($\bar{\text{x}}$ 21.4) (for *P*. *nemorale*) to even 17–34.1 μm ($\bar{\text{x}}$ 25.9) (for *P*. *longisetum*). The cell dimensions make the leaf cell aerolation more dense than in the previously mentioned species, and it looks more like in *P*. *cavifolium* [3, 9, 16, 18].

*Plagiothecium angusticellum* is a species whose current range is limited to Central Europe (the Czech Republic, Estonia, Hungary, Latvia, Lithuania, and Poland). In this area, it was listed in: *Ribeso nigri-Alnetum glutinosae*, *Fraxino-Alnetum*, *Luzulo pilosae-Fagetum*, *Tilio*-*Carpinetum*, as well as spruce and beech forests. Is these phytocenoses, it was recorded in the epigeic (mineral soil), epilithic (stones and rocks), and epiphytic habitats (*Acer* sp., *Alnus glutinosa*, *Quercus robur*, *Carpinus betulus*) (S1 Text).

## Discussion

Studies on intraspecific variability of *P*. *nemorale sensu lato* indicate that it is a complex including taxa that differ in both qualitative and quantitative characteristics [19–22].

Detailed type analysis (NY *913349*, PC*132573*, PC*132572*, H-SOL *1563011*) and analysis of protologues of synonyms of *P*. *nemorale sensu lato* [9–14] indicate that individual taxa differ in both qualitative and quantitative characteristics. We note differences, among others, in: turf color, leaf symmetry, as well as the shape and serration of the apex. That these features are taxonomically significant for *P*. *longisetum* were pointed out by Lindberg [9]. This author wrote that the turf of this species was pale greenish or yellow green, leaves in dry conditions usually gently shrunken, asymmetrical and oval, with a non-serrate margin. Nevertheless, the length and width of the leaf cells (and thus their shape) are the most important for species of *Plagiothecium* [3, 15–19, 21–23].

The cells from the central part of the leaf have always played the most important diagnostic role [3, 15–18, 23–24]. However, Wolski’s research [19, 21] shows that cells from other leaf zones are equally important from a taxonomic point of view. The above-presented studies confirm these results.

Although for the last 50 years, *P*. *longisetum* has been considered a synonym of *P*. *nemorale* [16, 28], our results indicate that they are separate species. *Plagiothecium nemorale sensu stricto* is characterized by wide and short cells (among others $\bar{\text{x}}$: LC2 96.7, WC2 22.2 μm), while *P*. *longisetum* has wide and long cells (among others $\bar{\text{x}}$: LC2 129.9, WC2 25.9 μm) (S3 Table). Lindberg [9] has already written about wide cells and lax cell areolation of *P*. *longisetum*. Also what is important and what the above-presented research shows, specimens of *P*. *longisetum* from Asia are similar to specimens from Europe.

Although *P*. *nemorale* and *P*. *longisetum* are listed in a similar area, the first of them seems to have a wider range of ecological amplitude. In addition, both were noted on other tree species, *P*. *nemorale* on: *Fagus crenata*, *Betula* sp. and *Quercus* sp., while *P*. *longisetum* on: *Acer* sp., *Fraxinus excelsior*, *Alnus glutinosa*. Moreover, *P*. *longisetum* was noted more often in epilithic and epixylic habitats (S1 Text). *P*. *angusticellum* has a much narrower geographical range than the previous two species (occurs only in Central Europe), and usually grows on bark of: *Acer* sp., *Alnus glutinosa*, *Quercus robur* and *Carpinus betulus* (S1 Text). The difference in ecological preferences of these taxa confirms previous observations of Wolski et al. [22] on the impact of overgrown habitat on variability of the *P*. *nemorale sensu lato*. However, it cannot be excluded that further detailed research will supplement our knowledge on this subject.

Recent articles about the genus *Plagiothecium* [4–7] supported by molecular analyses show a new point of view on the relationship between individual taxa of this genus. The genus is not only described as extremely variable but also comprises several dozen taxa awaiting detailed research and determination of their taxonomic status [4]. Our research confirms these observations, restoring one previously synonymous species and describing a new species.

To formulate a proper hypothesis about the phylogeny of *Plagiothecium*–a group which represents complexes of closely related taxa–the molecular differences among both nuclear and chloroplast regions should be investigated [5]. Therefore, we have analysed the nuclear ITS and chloroplast the *rps4* and *rpl16* genes because they are frequently sampled markers in bryophyte phylogenies, and the latter can yield a phylogenetic signal even at the lowest population level [35]. The analysis of only nuclear ITS (Fig 4) has confirmed that studied Wolski specimens belong to the complex of *Plagiothecium*. The proper molecular results, confirming morphological investigations, were retrieved when the analysis was expanded with chloroplast DNA markers. All Wolski specimens designated as *P*. *longisetum* belong to the clade which has been separated from *P*. *nemorale* (Fig 5).

Our results show that considerable morphological and genetic variation exists between *P*. *longisetum* and *P*. *nemorale*. Thus, the existing taxonomic, morphological and genetic differences are so unambiguous that they confirm the legitimacy of considering them as separate species. That is why we believe that our detailed analysis shows that *P*. *longisetum* should be recognized as a separate species; therefore, we propose to restore it.

In addition, molecular analyses supported by morphological differences give rise to the distinction a new species–*P*. *angusticellum* (Figs 5 and 6). Morphometric data confirm phylogenetic moieties, providing easily distinguishable diagnostic features. Morphological differences and phylogenetic relationships indicate that these groups are recognizable and represent independent lines of evidence that support their recognition as separate species.

Features that distinguish *P*. *longisetum* from *P*. *angusticellum* are shrunken leaves, the width of the leaf, the shape and curvature of the leaf apex, the length of costae, the length and the width of the leaf cells, irregular rows of cells, delicate cell asymmetry and tight cell areolation (Figs 2 and 6, S4 Table, Table 4).

**Table 4:** Comparison of the main diagnostic features of the described species.

Features	*P*. *longisetum*	*P*. *angusticellum*
**Shrunken leaves**	yes	no
**Width of the leaf [mm]**	1.6–2 ($\bar{\text{x}}$ 1.7)	1.3–1.5 ($\bar{\text{x}}$ 1.4)
**Shape of the leaf apex**	acute to acuminate	acuminate
**Curvature of the leaf apex**	straight	often gently curved
**Length of costae [mm]**	1.1–2.4 ($\bar{\text{x}}$ 1.5)	0.7–1.4 ($\bar{\text{x}}$ 1.1)
**Leaf cell**	long and wide	long and narrow
**LC1 × WC1 [μm]**	68.5–158.1 ($\bar{\text{x}}$ 104.9) × 17–32.3 ($\bar{\text{x}}$ 24.7)	81.7–120.4 ($\bar{\text{x}}$ 103.7) × 13.2–19.5 ($\bar{\text{x}}$ 16.7)
**LC2 × WC2 [μm]**	94.6–150.3 ($\bar{\text{x}}$ 129.9.0) × 17–34.1 ($\bar{\text{x}}$ 25.9)	113–143.3 ($\bar{\text{x}}$ 125.9) × 15.1–19.3 ($\bar{\text{x}}$ 17.4)
**LC3 × WC3 [μm]**	69.1–223.1 ($\bar{\text{x}}$ 159.1) × 19.9–40.2 ($\bar{\text{x}}$ 29.3)	123.1–172 ($\bar{\text{x}}$ 140.8) × 16.4–24.6 ($\bar{\text{x}}$ 20.3)
**Irregular rows of cells**	no	yes
**Delicate cell asymmetry**	no	yes
**Tight cell areolation**	no	yes

## Supporting information

S1 Text Specimens of *Plagiothecium nemorale*, *P*. *longisetum* and *P*. *angusticellum* examined. The numbers in square brackets refer to individual specimens in the file S1 Raw Data.
(DOC)

S1 Raw Data (XLSX)

S1 Table Summary of partitions for evolutionary model selection and phylogenetic interference using PartitionFinder2. A: partitions for ITS matrix (710 bp) analysis; B: ITS-*rps4*-r*pl16* matrix (2068 bp) analysis.
(DOCX)

S2 TableDescriptive statistics of the examined specimens of *Plagiothecium nemorale sensu lato*.LC1, LC2, LC3, WC1, WC2, WC3 –explanation in Table 1; N–number of observations, $\bar{\text{x}}$ –mean, Me–median, Min–minimum, Max–maximum, Q1 –first quartile, Q3 –third quartile. Data ($\bar{\text{x}}$, Me, Min, Max) are given in μm.(DOC)

S3 TableDescriptive statistics of individual characteristics of the examined species.LC1, LC2, LC3, WC1, WC2, WC3 –explanation in Table 1; N–number of observations, $\bar{\text{x}}$ –mean, Me–median, Min–minimum, Max–maximum, Q1 –first quartile, Q3 –third quartile. Data ($\bar{\text{x}}$, Me, Min, Max) are given in μm.(DOC)

S4 TableDescriptive statistics of individual features of the studied taxa.LC1, LC2, LC3, WC1, WC2, WC3 –explanation in Table 1; N–number of observations, $\bar{\text{x}}$ –mean, Me–median, Min–minimum, Max–maximum, Q1 –first quartile, Q3 –third quartile. Data ($\bar{\text{x}}$, Me, Min, Max) are given in μm.(DOC)

S5 Table The results of the Student’s t-test analysis together with the effect measure (Cohen’s d) for the *P*. *longisetum* and *P*. *angusticellum*. (DOC)

S6 Table Genetic distance between *Plagiothecium* taxa based on ITS*-rps4*-*rpl16* matrix. Highlighted the distance between *P*. *nemorale*, *P*. *longisetum* and *P*. *angusticellum* specimens (asterisk indicate specimens described as *P*. *angusticellum*).
(DOCX)

S7 Table Genetic distance between *Plagiothecium* taxa based on ITS part of the matrix. Highlighted the distance between *P*. *nemorale*, *P*. *longisetum* and *P*. *angusticellum* specimens (asterisk indicate specimens described as *P*. *angusticellum*).
(DOCX)

S8 Table Genetic distance between *Plagiothecium* taxa based on *rps4* part of the matrix. Highlighted the distance between *P*. *nemorale*, *P*. *longisetum* and *P*. *angusticellum* specimens (asterisk indicate specimens described as *P*. *angusticellum*).
(DOCX)

S9 Table Genetic distance between *Plagiothecium* taxa based on *rpl16* part of the matrix. Highlighted the distance between *P*. *nemorale*, *P*. *longisetum* and *P*. *angusticellum* specimens (asterisk indicate specimens described as *P*. *angusticellum*).
(DOCX)

S1 Fig Distributions of variables for cell length (A) and width (B) *Plagiothecium nemorale sensu lato*. The values of the x axis are given in μm.
(DOC)

S2 Fig Distributions of variables for individual species. Red line–*P*. *nemorale sensu stricto*, green line–*P*. *longisetum*. The values of the x axis are given in μm.
(DOC)
