# Supplementary material for: Resurrection of the Plagiothecium longisetum Lindb. and proposal of the new species—P. angusticellum
Source: PLoS One. 2020 Mar 11;15(3):e0230237. doi: 10.1371/journal.pone.0230237 (PMC7065767; doi:10.1371/journal.pone.0230237)
Supplement: S1 Text — The numbers in square brackets refer to individual specimens in the file S1 Raw Data. (DOC) [file pone.0230237.s001.doc]

**S1 Text specimens of *Plagiothecium nemorale*, *P*. *longisetum* and *P.* *angusticellum* examined.** The numbers in square brackets refer to individual specimens in the file S1 Raw Data.

***Plagiothecium nemorale***

*Plagiothecium nemorale* (Mitt.) A.Jaeger, Ber. S. Gall. Naturw. Ges. 1876-1877: 451 (1878) ≡ *Stereodon nemoralis* Mitt., Journ. Linn. Soc. Bot. Suppl. 1: 104 (1859) ≡ *P*. *silvaticum* var. *nemorale* (Mitt.) Par., Ind. Bryol.: 967 (1898). Type: In Himalayae orient. reg. temp., Sikkim, in monte Tonglo (ad radicem filicis cujusdam), sine dato, *J. D. Hooker s.n.* (NY 913349) = *P*. *neglectum* Mönkm. Laubm. 866 (1927). Type: sine loco, sine dato, *sine coll.* *s.n*. = *P*. *saxicola* Sak., Bot. Mag. Tokyo 48: 395 (1934). Type: Hondo, Prov. Aki, Mt. Fukuȏji, ad saxas, 4 Jan 1933, *Y. Doi Typus in Herb. K. Sakurai 3282* (PC132573) = *P*. *silvaticum* var. *latifolium* Cardot, Bull. Bot. Soc. Genève sér. 2, 4: 385 (1912). Type: Japon, Iyo (Gono). Corée: île Quelpaert, Faurie *507* = *P*. *silvaticum* var. *rhynchostegioides* Cardot, Bull. Bot. Soc. Genève sér. 2, 4: 385 (1912). Type: Japon: Mororan, bas-fonds, sur pierres, *Cardot 2965*.

**AUSTRIA**: Ternovanerwald prope Görz, in trunco putrido, alt. 1100 m, Oct, *C*. *Loitlesberger*, BM*1007967* [169]. **BELGIUM**: Louette-saint-Pierre, rochers ombragés, 10 Aug 1882, *F*. *Gravet*, BM*1007970* [170]. **CHINA**: Hanzghou County, Tinmu Mountain Nature Reserve, West Tianmu Mt., 30°50ʹ N, 119°25ʹ E, alt. 400-1000 m, subtropical broadleaf evergreen forest, with *Cryptomeria fortunei* and *Ginko biloba* and extensive granitic outcrops, 26 Aug. 1993, *W*. *R*. *Buck*, *23865*, NY *3103645* [207]; Liaoning Prov., Kuandian County, Bai-shi-la-ji, Mountain, Bai-shi-la-ji National Natural Reserve, trail from fire tower to Xiang-shui-gou at Hu-li Mt., 40°50ʹN, 124°52ʹE, alt. 900–1000 m, moist hardwood forest with numerous granitic boulders, 21 Aug 1993, *W*. *R.* *Buck*, *23747*, NY *3103644* [206]. **CZECH REPUBLIC**: Plzeň jih, Ždírec u Plazně, Chejlava, na zemi v bučině v přírodní rezervaci, alt. 620 m, 8 Jun 1974, *M*. *Vondráček*, PL *169/382* [11]; Rýmařov, Valšovský Žleb, asi 1.7 km from Sovinec, 22 Sep. 1975, *R*. *Doležal*, SNM *161* [4]; Šumava, Zilená Lhota, u potoka 1.5 km JJV od železniční stanice, alt. 640 m, 27 Aug 1984, *M*. *Vondráček*, PL *169/586* [9]. **DENMARK**: Allindelille skov, on tree stump, 31 Mar 1988, *H*. *Nielsen*, C-M-*20159* [148], på stød, alt. 40 m, 31 Mar 1988, *H*. *Nielsen*, C-M-*20147* [145]; Bornholm, Alindinge, På fugtig jorddækket klippevæg nær ved Borre Sø, 16 Dec 1981, *S*. *Henriksen*, *81*-*169*, C-M-*20145* [146]; Draved Skov ved Løgumkloster (på træstub), 28 Mar 1951, *K*. *Holmen*, C-M-*20139* [151], Nørlund Skov, 18 Oct 1971, *K*. *Holmen*, C-M-*20144* [152]; Stensves Skov, alt. 39, 5 May 1951, no collector C-M-*20233* [149]. **ESTONIA**: Pärnu mk. Kiigemäe, lodumets ranna ääres, tüvealusel, Jul 2000, *K*. *Vellak* TAA *5004963* [52]; Tartu, Siniküla, 29 Apr 2003, *K*. *Vellak* TAA *5004961* [51]; Saare maakond, Kaarma vald, Abruka saare keskosa, Iaialehine salumets tüvealune, 58°8ʹ25ʺN, 22°31ʹ15ʺE, 6 Jul 2011, *L*. *Ehrlich* TAM B *874:42* [54]. **FRANCE**: Larran, Basses Pyrenees, in extensive sheet on wet vertical face of basic rock in deep shade, alt. 700 m, 16 Apr 1952, *A*. *C*. *Crundwell*, E*905535* [75]; Eure, Les Andelys, bois Renard, 31 Mar 1904, *F*. *Camus*, PC*22845* [174]; Forêt de Fontainebleau, Gorgea aux Loups blocs degrés, 14 Apr 1912, *F*. *Camus*, PC*22852* [172]; Haute Saone, Faucogney, an nassen Hornfelsklippen oberhald der Kapelle von Breuchin, alt. 400 m, 23 Mar 1988, *J*.-*P*. *Frahm*, C-M-*20101* [156]; Saint Yon, Essonne, Bois de Baville, sur talus ombragé de chemin creux, 7 Nov 1992, *O*. *Aicardi*, PC*31331* [171]. **GEORGIA**: Kobuleti, in valle rivi Ckheni in vicinitate oppidi Chakva, 100-600 m, 29 Jul 1986, *V*. *Vašák*, NY *3103752* [89]; Sukhumi distr., in vicinitate pagi Omarishara, in valle rivi Klich, alt. 1.300–1.500 m, 6 Jun 1986, *V*. *Vašák*, NY *33684222* [86]. **GERMANY**: Bavaria, Berchtesgaden district, Schleching, on the ground in a wood, 9 Jun 1962, *S*. *S*. *Hooper*, E*905518* [98]; Rheinland-Pfalz, Kreis Cochem, Naturschutzgebiet, Dortebachtal, along trail, from parking area to waterfall, ca. 1 km E of village of Klotten along, Hwy 49, along N shore of Mosel River, 50°10ʹ07ʺN, 7°12ʹ48ʺE, alt. 150 m, steep, narrow valley along small stream (Dortebach) with mixed hardwoods, 30 Jan 2010, *W*. *R*. *Buck 55948*, NY *1167658* [99]. **HUNGARY**: Borsod, in peteosis porphyricis silvat., supra vallem Felsősebes, prope Ómassa, alt. 650 m, 7 Oct 1952, *A*. *Boros*, BRNU *648207* [16], in rupibus calc. umbr. cunicli “Udvarkó” prope Lillafüred, alt. 430 m, 22 Jun 1932, *A*. *Boros*, BRNU *294* [18], in rupibus umbrosis calcareis vallis Leány-völgy prope Nagyvisnyó, alt. 700, 7 Aug 1953, *A*. *Boros*, BRNU *648210* [17]; Esztergom, in rupibus andesiticis umbrosis vallis Nyir-völgy prope Pilismarót, alt. 260 m, 24 Aug 1952, *A*. *Boros*, BRNU *648203* [22]; Fejér, ad donten Dobaikut pr. pag. Oroszlány, mtes Vértes, 20 Jul 1954, *L. Vajda*, H*3113604* [29]; Komárom, in alnetis ad fontem Dobai-kut in valle rivi Fekete-Ér prope Oroszlány, alt. 220 m, 20 Jul 1954, *A*. *Boros*, BRNU *648212* [20], in loc. Humidis silvat. Ripae rivi “Mocsár-berek” pr. Oroszlány, alt. 250 m, 8 Sep 1937, *A*. *Boros*, BRNU *165* [21], in locis humidis silvat. Ripae rivi vallis Bodony-völgy prope Oroszlány, versus fontem Buger-kut, alt. 200 m, 21 Jun 1937, *A*. *Boros*, BRNU *169* [19], in sylvestribus ad fontem Dobaikut prope pag. Oroszlány, 20 Jul 1954, *L*. *Vajda*, H*3113603* [26]; Pest, in rupibus andesit, silvat., partis superioris vallis rivi Bükköspatak, prope Pilisszentkereszt, alt. 400 m, 3 Aug 1952, *A*. *Boros*, BRNU *648199* [23]; Vas, ad latera torrentis silvat., vallis, Hárompatakvölgy, prope Köszeg, versus pag. Hámortó, alt. 400 m, 9 Sep 1930, *A*. *Boros*, BRNU *276* [24]; Veszprém, ad margines rivi in sylvestribus prope pag. Márkó, montes Bakony, 1 May 1967, *L*. *Vajda*, H*3237546* [27]; Zala, in alnetia ad Határ-folyás propr Kisrada, alt. 100 m, 22 Mar 1952, *A*. *Boros*, BRNU *548218* [25]. **IRAN**: Mazandaran province, Nowshahr country, lower part of Veisar forest, alt. 1170 m, 2009, *H*. *Zare*, S B*171129* [183], lower part of Veisar forest, *Fagus*-*Carpinus* forest type, 890 m, 2009, *H*. *Zare*, S B*171134* [185], Jamand forest, alt. 1350 m, 2009, *H*. *Zare*, S B*171132* [184]. **INDIA**: no location and collector, E00905551 [208]. **ITALY**: Veneto, Mt. Canzeglio, bei Vittorio auf Baumstümpfen, 25 Jul 1895, *M*. *Fleischer*, C-M-*20081* [168]. **JAPAN**: Kagoghima Pref., Yakushima Island, alt. 420 m, on moist vertical face of boulder in full shade, 24 Sep. 1975, *Z*. *Iwatsuki*, *G*. *L*. *Smith*, *J*-*936*, NY *3103681* [216]; Prov. Sôya, Isl. Rishiri, Mt. Rishiri, alt. 100-500 m, 8 Aug. 1954, *H*. *Hasegawa*, *Z*. *Iwatsuki*, *15944*, H*3113590* [123]; Hokkaido, Rishiri Island, Mt. Rishiri, alt. 500 m, 8 Aug 1954, *H*. *Hasegawa*, *15783*, H*3113589* [125]; Honshu, Gifu-ken, Mugi-gun, Itadorimura, Otani valley, alt. 300–400 m, on tree trunk, 26 Oct 1987, *M*. *Mizutani*, *13172*, NY *3103647* [213], 29 Oct 1987, *M*. *Mizutani*, *13172*, S B*281901* [191], Mie Pref., Ihadaka-cho, Myojindaira on trunk of *Fagus crenata*, alt. 1280 m, 17 Aug 1976, *T*. *Magofuku*, S B*281908* [186], Miyajima Isl., Hiroshima Pref., on soil, 28 Apr 1968, *H*. *Ando*, H*3113620* [127], Mt. Hibayama, Hiroshima Pref., alt. 1200 m, on rock, 11 Oct 1973, *H*. *Ando*, S B*281902* [190], Mt. Kiso-Ontake, on rocks in conifer forest, alt. 1250 m, 24 Aug 1953, *N*. *Takaki*, H*3113623* [126], Ibazi Prov., 27 Oct 1921, *R*. *Jakenekin*, H*3113587* [124]; Kiushiu, center of back bone (Grup of Mt. Kumimi), Gakagoe, Kumamoto flank, humus, alt. 1200, 13 Aug 1951, *Y*. *Kuwahara*, NY *3103653* [215], Pref. Kumamoto, Mt. Fukaba, 22 Jul 1934, *N*. *Takagi*, S B*281907* [188]; Nara Pref., Simokitayama-mura, alt. 900 m, 12 Aug 1971, *T*. *Kodama*, *43536*, E*905532* [78]. **LATVIA**: Salacgriva, district, Vecsalaca forestry, wet drained spuce forest, on crystalline stone, 57°44ʹ18ʺN, 24°27ʹ48ʺE, 13 Jul 1962, A. *Ȃbolina* SILAVA *4576* [68]. **LITHUANIA**: Alytaus raj., Vizgiris, Ant beržo kelmo, 0.5 m aukščio nuožemiės, šiaurinėje pusėje, 13 Jul 1971, *Z*. *Kuzas*, BILAS *5624* [62]; Biržų raj., Biržų giria, Biržų girios bot. dr. 202 kv. 16 skl. Ant pŭvančios medienos, 13 Jul 1998, *I*. *Jukonienė*, *R*. *Radzevičŭtė*, BILAS *10621* [61]; Kaišiadorių raj., Rumšiškių km., Upelio pakr., mišriame miškas, 17 Aug 1956, *J*. *Pipinys*, BILAS *5125* [67]; Lazdijų r., Metelių apyl., Metalių RP., Širvinto miškas, 6 kv., 1 skl., Šlaite, 22 Aug 2003, *I*. *Jukonienė*, BILAS *5722* [65]; Marjampolės, r., Žaltyčio g-ja, Buktos miškas, 55 kv., 5 skl., Lapuočių miške, 17 Aug 1995, *V*. *Marozas*, BILAS *B9346* [57]; Radviliško raj., Radvilonių miškas, ant išversto medžio šaknų, 24 May 1984, *I*. *Liepinaitytė*, BILAS *4122* [66]; Varėnos r., apie 3 km i Š nuo Merkinės, Mikalauciškės k., Dzŭkijos nacionalinis parkas, Ežero šlaito ąžuolų miške, 19 Aug 1998, *A*. *Skirpastas*, BILAS *B6427* [60]; Vilkaviškio r., Paržerių g-ja, Uosijos miškas, 11 kv., 14 skl., Drėgname uosyne, 3 Sep 1998, *R*. *Čiuplys*, BILAS *B6418* [59]; Vištytgiris r., Pajevonio g-ja, Draustgirio miškas, 35 kv., 1 skl., Drausgirio gamtinis rezervatas, Vištyčio regioninis parkas, Eglių ir skroblų miške, 16 May 1998, *R*. *Valatkevičius*, BILAS *B3696* [64]. **NORWAY**: Hordaland, Bømlo, Berg ogur ved klebersteinsbrotet, 59,7048° 5,1765°E, 10 Apr 2016, *A*. *Knutsen et al.,* TRH B-*12771* [135]; Rogaland, Ursira, 24 Jul 1916, *R*. *Nordhagen*, BG B-*30484* [133], Vindafjord, Opsal, Vikedal, Edellauvskog, alt. 60 m, 59,5025°N 5,8746°E, 17 Oct 2015, *A*.*H*. *Øygarden et al.*, TRH B-*12753* [136]. **POLAND**: Nadleśnictwo Balinka, Puszcza Augustowska, łęg, 5 Sep 1963, *D*. *Gocławska*, IBL *1437* [32]; Białystok okolice uroczyska Antoniuk, nadl. Dojlidy oddz. 120, łęg, 26 May 1988, *A*. *Sokołowski*, IBL *8671* [33]; Rezerwat Rozpuda, bór świerkowy torfowcowy, 25 Jun 1986, *A*. *Sokołowski*, IBL *8314* [30]; Wigierski Park Krajobrazowy, nad. Maćkowa Ruda, *Circaeo-Alnetum*, oddz. 186, 19 Jun 1983, *A*. *Sokołowski*, IBL *7507* [31]; Karpaty, Beskidy, Beskid Śląski Mts., rezerwat Wisła, dolina potoku Biała Wisełka, na skale, na piaskowcu, 2002, *A*. *Stebel*, SOSN *42215* [38]; Wielka Puszcza, *A*. *Stebel*, SOSN *57336* [40], Beskid Śląski, Szyndzielnia Mts., gleba, skarpa, 2000, *A*. *Stebel*, SOSN *34993* [39]; Bory Tucholskie National Park, *S*. *Lisowski*, POZG-B *12818* [37]; Poj. Gnieźnieńskie, Kąty, gm. Murowana Goślina, buczyna, na glebie przy pniu dębu, 30 Apr 1988, *W*. *Bocheński*, POZG-B *7126* [34]; Świętokrzyski Park Narodowy, gleba w lesie bukowym, oddz. 67a, ATMOS Ee76, alt. 339 m, 4 Aug 2012, *T*. *Paciorek*, KRAM B *224549* [47]; West Pomerania, Buczyna Wąwozy nature reserve, *E*. *Fudali*, KRAM B *129290* [48], Kołowskie Parowy nature reserve, *E*. *Fudali*, KRAM B *129365* [49]. **PORTUGAL**: Coimbra, Luso, Bussaco, Cruz Alta viewpoint-E Sáo Miguel, Palace Hotel, alt. 370-500 m, soil, 22 Sep. 2000, *L*. *Hedenäs*, S B*44516* [217]. **SLOVAKIA**: Little Farta, Stankovany Rezervace Slatiny, *Z*. *Pilous*, SNM *228* [14]; Mony, Malá Fatra, *Z*. *Pilous*, BRNU *343116* [12]; Slovensky Raj, Čingov Sokolia Dolina, *Z*. *Pilous*, SNM *229* [15], Stratena u Dobšinsjé, *Z*. *Pilous*, SNM *229* [13]. **SWEDEN**: Blekinge, Tving par., Alnaryd, , 28 Jul 1888, *H*. *W*. *Arnell*, UPS B-*601052* [165]; Halland, Simlangsdalen, Klyvaberg, *Fagus silvatica* wood, 22 Oct 1966, *K*. *Holmen*, C-M-*20127* [160]; Småland (Inre), Växjö par., Härlöv, 6 Oct 1970, *I*. *Söderberg*, UPS B-*601064* [162], Småland (Kalmar), Oskarshamn par., Oskarshamn, bortom, stadsparken, Sep 1928, *L*. *Söderberg*, UPS B-*601065* [161], Agunnaryd, 5 Jul 1962, *J*. *Christoffersson*, S B*108962* [222]; Södermanland, Botkyrka sn., on rock on the south-steep of Örnberget at Tullinge, 3 Sep 1967, *E*. *Nyholm*, *G*. *Een*, S B*86340* [224], Värmland, Boda, Renstadsnipans, sydbrant, bergvägg, alt. 500 m, Bergvägg, 14 Sep 1995, *S*. *Fransson*, S B*106240* [218]. **TURKEY**: Giresun Prov., wet soil-cutting beside the road Giresun-Ordu, about 4 km E of Bulancak, alt. 20 m, 5 Nov 1974, *T*-*B*. *Engelmark* & *E*. *Nyholm*, S B*68171* [199]. **UNITED KINGDOM**: Alderley Edge, waterfall, wood by edge of stream in valley, 16 Mar 1997, *S*. *Edwards*, MANCH Kk *1583* [202]; England, Abundant on soil in small copse by bridle way E of road between East Horsley and Mountain Wood, Surrey, v-c. 17, 20 May 1978, *C*. *C*. *Townsend*, *78/429*, E*905445* [118], Flatford, East Bergholt, Suffolk, Steep roadside bank, 7 Mar 1958, *D*. *H*. *Dalby*, *3201*, BM*1103858* [204], Cambridgeshire, Hayley wood, oak base, Nov 1948, *P*. *J*. *Chamberlain*, *677*, E*350155* [116], East Kent, near Bredhurst, 7 Apr 1967, *S*. *A*. *McGregor*, E*242675* [117]; Smallbrook valley, Surrgy, on bark, 18 Apr 1987, *S*. *R*. *Edwards*, MANCH Kk *1939* [201]; Loch Lomond, on rock, 17 May 1953, *A*. *C*. *Crundwell*, E*905494* [106], Stirlingshire, 17 May 1953, *A*. *C*. *Crundwell*, E*905493* [109]; Campsie Glen, Stirlingshire, 28 Feb 1959, *A*. *C*. *Crundwell*, E*905495* [108], Stirlingshire, 11 Mar 1950, *A*. *C*. *Crundwell*, E*905492* [107]; Isle of Arran, Carre Caves, New Red Sandstone, 23 Mar 1964, *H*. *J*. *B*. *Birks*, *25443*, E*422682* [110]; Inverneil Burn, Knapdale, on bank in gorge, 6 Sep 1966, *M*. *F*. *V*. *Corley*, E*905482* [111]; Scotland, on sandy soil in the shade of a rock, S end of the Isle of Skye, just above the Sleat Peninsula, N. Ebudes, v-c., 104. 23 Aug 1968, *C*. *C*. *Townsend* E*905483* [112], North Ebudes, Rum, East of Sahmnan Insir, Rubha na Moine, grassy ledge at base of sea cliff, date unknown, *D*. *F*. *Chamberlain*, E*110158* [113]; West Borfolk, near Beetley, wooded heath, on shady log, v-c. 28, alt. 10 m, 13 Apr 2003, *D*. *G*. *Long*, *31916*, E*905439* [115]; Worcestershire, N slope of Bredon Hill, above Woolas Hall, *Fraxinus* woodland, on soil bank under trees, v-c. 37, alt. 220 m, 4 Apr 2004, *D*. *G*. *Long*, *33099*, E*905436* [114].

***Plagiothecium* *longisetum***

*Plagiothecium longisetum* Lindb., Acta Soc. Sci. Fenn. 10: 232 (1875). Type: Japan. ad Nikosan ins. Kiusiu, fertile, 16 Junii 1863, *S.O. Lindberg* (PC*0132572*, H-SOL *1563 011*) = *P*. *longisetum* var. *brevinerve* Ihsiba, Trans. Sapporo Nat. Hist. Soc. 13: 396 (1934). Type: Japan. Mt. Hakkȏda, Mutsu, 1933, *S*. *Murai* *s*.*n*.

**AUSTRIA**: Auf Waldboden bie Wolfart schlag zwischen Sankt Anton und Frankenfels,
1 Aug 1877, *J*. *B*. *Förster*, E*905511* [70]. **AZORES**: Flores, 1937, *H*. *Persson*,
S B*43144* [196]; Pico, May 1937, *H*. *Persson*, NY *3103691* [93]; Terceira, central part, Terra Brava, alt. 700 m, soil-covered rock, 3 Oct. 2000, *L*. *Hedenäs*, S B*42785* [195]. **BELGIUM**: Commune de Yvoir, aux confins de Dorinne, Durnal et Purnode, vallée du Bocq, dans un bois de pente á exposition sud, sur rochers, Apr 1979, *J*. *L*. *DeSloover*, *30*.*246*, NY *3103766* [71]; Meix-devant-Virton, sortie vars Houdrigny, carriere abandonnée au bord de la route, sur paroi de calcaire sinémurien, 19 May. 1981, *J*. *L*. *D*. *S*. *Namur*, *34188*, C-M-*20116* [144]. **CHINA**: Diqing prefecture, Weixi County, W side of Litiping Plateau, 27°09ʹ07ʺN, 99°24ʹ36ʺE, ravine in degraded *Acer*/bamboo mixed forest, on shady tree base, alt. 3130 m, 19 Jun 1993, *D*. *G*. *Long*, *24527*, E*7047* [74]; Gong-Shan County, Gaoligong Shan Range, southern end of the Hengduan Shan, Nu Jiang (Salween River) watershad. Below Dulong Road, along tributary of Pula He near Daladi site, 41 km from Gongshan, 27°47ʹ38.4ʺN, 98°30ʹ29ʺE, alt. 2950 m, open mixed hardwood-conifer forest of *Acer*, *Abies* and scattered *Larix*, on granitic boulder and *Acer* roots, 4 Oct 2002, *J*. *R*. *Shevock*, *23371*, E*844311* [72], open mixed hardwood-conifer forest of *Acer*, *Abies* and scattered *Larix*, on damp shaded granitic rock wall, 4 Oct 2002, *J*. *R*. *Shevock*, *23356*, E*844310* [73]. **DENMARK**: Draved Skov ved Løgumkloster (på træstub), 10 May 1971, *J*. *Lewinsky* C-M-*20140* [153]; Sjaelland, St. Hedding, dist. 39b, 19 Sep. 1954, *I*. *Gernå*, C-M-*20201* [150], St. Møsten Skov, på skovbund, 23 Apr. 1983, *S*. *Henriksen*, *83*-*010*, C-M-*20146* [147]. **ESTONIA**: Ida-Viru, Tudu, 12 Aug 1996, *M*. *Leis* TU *151281* [53]. **FINLAND**: Alandia, Föglö, Degerby village, on stonnes and humus soil in hazel grove, in Gripö Island, 21 Aug. 1968, *P*. *Karlström*, C-M-*20120* [154]. **FRANCE**: Südvogesen, Bachschlucht S La rosiere, ca. 15 km, S Remiremont, Granitgestein, alt. 520 m, 23 Sep 1981, *Eggers* & *Frahm*, NY *3103761* [76]; no locality, *Kleinhans*, THR B-*88905* [122]; Deux-Sèvres, Vernoux, Ruisseau des Grinchères, Jul. 1963, *P*. *Biget*, *24796*, PC*7774059* [177]; Forêt de Compiègne, Baltigny, talus sablonneux, 9 Jun 1912, *F*. *Camus*, PC*22846* [173]; Seine-et-Marne, Orly-sur-Morin, bois du Petit, Villiers, 19 May 1912, *F*. *Camus*, PC*22833* [176]; Seine-et-Oise, forêt de Marly, 19 Mar. 1916, *F*. *Camus*, PC*22835* [175]; Haute-Saine, Suedwestvogesen, Ruisseaux de Ballon E Haut le Rhan bei der Refuge Fray, kalkspatreicher Granit, alt. 700 m, 3 Oct. 1999, *J*.-*P*. *Fram*, C-M-*20098* [155]; No locality, *Kleinhans*, THR B-*88905* [122]. **GEORGIA**: Adzharia, Botanic Garden, Batumi, on sandy bank under hedge, 9 May 1988, *C*. *C*. *Townsend*, E*905554* [100]; Reseratum Lagodechi, in vicinitate oppidi Lagodechi, alt. 600 m, 28 May 1987, *V*. *Vašák*, NY *3684221* [82]; Sukhumi distr., latior pagi Pskhu, in valle fluminis Bzyb, dextra inter rivos Aguripsta et Pchica, alt. 400–440 m, 15 Aug 1983, *V*. *Vašák*, NY *3684218* [84]. **GERMANY**: Allgau, Sauwald ob Hinterstein auf rethern Hornstein, alt. 1000 m, 19 Aug. 1890, *A*. *Holler*, C-M-*20085* [157]; Badem-Württemburg, Headwaters of the Elz River near Korallenhäusle, NW of Schönwald, NE part of Franberg, Naturpark Südschwarzwald, alt. 927 m, on rock in stream, 24 Apr. 2010, *J*. *T*. *Wynns*, CP-*10635* [158]; Badem-Württemburg, Mooswald, a mesic riparian forest beside autobahn (5/E35), near Tiengen, W of Freiburg, Kaiserstuhl region, 47°58ʹ56.5ʺ N, 7°44ʹ21ʺ E, alt. 210 m, growing over deached bark of huge rotten stump, 19 Apr. 2010, *J*. *T*. *Wynns*, CP-*10630* [159]. **INDIA**: Kashmir, Gulmarg, 26 Jul 1926, *R*. *R*. *Stewart*, *8677*, NY *3103685* [97]. **IRAN**: Mazandaran province, Nowshahr country, lower part of Veisar forest, alt. 840 m, 2009, *H*. *Zare*, S B*171133* [181], alt. 1300 m, 2009, *H*. *Zare*, S B*171130* [182], Mashlak to Veisarforest, alt. 660 m, 2009, *H*. *Zare*, S B*171131* [180]. **JAPAN**: Kasugayama, Nara, 6 Apr 1972, *H*. *Ando*, E*905546* [79]; Kyōto Pref., Bessyo, Hanase, Keihoku, chō, alt. ca. 650 m, on rock, 14 Sep 1975, *T*. *Kodama*, *51007*, E*905548* [80]; Gifu Pref., Mt. Ontake, vicinity of Nigori-daki Power Station, alt. 1500 m, on moist rotten log in full shade, 31 Aug. 1975, *Z*. *Iwatsuki*, *G*. *L*. *Smith*, *J*-*576*, NY *3103682* [212]; Hokkaido, Hidaka, Samani-ch, alt. 50 m, 20 Sep. 1970, *Kobayashi*, *Iwatsuki*, S B*281906* [187], alt. 20 m, 24 Sep. 1970, *Kobayashi*, *Iwatsuki*, S B*281904* [189]; Kiushiu, Siuyahakei, ground, 28 Apr. 1950, *Y*. *Kuwahara*, NY *3103654* [214]; Miyazaki Pref., Kobeze-no-taki, on boulder, 24 Jun. 1980, *M*. *Glime*, *Z*. *Iwatsuki*, NY *3103665* [211]; Shikoku, Mt. Ishizuchi, Ehime Pref., alt. 700 m, on decayed trunk, 1 Nov. 1971, *H*. *Ando*, H*3113621* [128]; Toyama, on stone, 29 Jun. 1928, *H*. *Jasaoka*, H*3113584* [129]. **MADEIRA**: Lavenda do Furado c. 1.5 km E of Riberio Frio, on loamy banks, alt. 750 m, 20 May 1996, *C*. *C*. *Townsend*, E*905556* [92]; 300 m, 11 May 1958, *L*. *F*. *H*. *Merlow*, E*905545* [91]; Location unknown, on soil, alt. 300 m, 11 May 1958, *L*. *F*. *H*. *Merlow*, E*905544* [90]; Along the levada between Ribeiro Frio and Lombo Capitâo Mormo, alt. 1.050-900 m, 25 Jun. 1952, *H*. *Persson*, *L*. *Hedenäs*, S B*8846* [193]; Ribeira do Joâo Fernandes, alt. 400-750 m, 4 Jun. 1952, *H*. *Persson*, *L*. *Hedenäs*, S B*8877* [192]; Sao Vicente, Rocha do Folhadal, alt. 1000-1100 m, epigeic, 30 May 1991, *L*. *Hedenäs*, S B*8885* [194]. **NEPAL**: Ghode Pani, c. 16 km NW of Pokhara, on ground in *Rhododendron* forests, 9 Nov 1978, *F*. *G*. *Davies*, E*905553* [94]; Taplejung District, Ridge NE of Gupha Pokhari, 27°17ʹN, 87°32ʹE, *Rhododendron arboreum* forest, on shady bank by stream, alt. 2775 m, 25 Oct 1991, *D*. *G*. *Long*, *21559*, E*2318* [95]. **NORWAY**: Hordaland, Bømlo, På berg i edellauvskog, 59,6358° N 5,2016° E, 9 Apr. 2016, *A*. *Knutsen* *et al.* TRH B-*153035* [140], Spyssøya, ves Myra, På bark av ustyva ask i beitemark, host: *Fraxinus excelsior*, alt. 48 m, 25 May 2014, *J*. *B*. *Jordan*, *H*. *H*. *Blom,* TRH B-*4712* [137]; Hordaland, Os, Bjørnen, 8 Oct. 1972, *D*. *O*. *Øvstedal*, *O*. *Balle*, BG B-*30525* [132]; Sør-Trøndelag, Frøya, Flatval, V for Husvatnet, Berg, alt. 20 m, 22 Aug 1985, *A*. *A*. *Frisvoll*, TRH B-*25795* [141], Bjugn, Dueskar, mot Stjørnfjorden, Rikt kratt, 6 Jun 1973, *A*. *Skogen*, BG B-*1547* [131]; Fredrikstad, Rauer, Bogenlia NR, På rikere bergvegg i edelløvskog, alt. 38 m, 8 Jun 2013, *T*. *Høitomt*, *K*. *A*. *Lye*, TRH B-*36977* [138]; Sverige, Västergötland, Alingsås, 5 Oct. 1913, *E*. *P*. *Vrang*, TRH B-*17301* [139]; Møre og Romsadal, Aure, Ånesstranda, Nordvendte kystberg, på berg, alt. 10 m, 9 Apr. 2007, *F*. *Oldervik* *et al.* TRH B*-671912* [134]; Rogaland, Rennesøy, 17 Jun 1971, *A*. *A.* *Frisvoll*, TRH B-*25780* [142]. **POLAND**: Poznań, uroczystko Olszak **na E od Jez.** Maltańskiego, lasy komunalne, oddzi. 39 m., las o charakterze łęgu olszowego z udziałem jesionu, na korzeniach olszy, 28 Sep 2002, *A*. *Rusińska*, POZG-B *7630* [35]; Pomorze Zachodnie, pow. Chojnice, okol. Upiłki, Żródliskowa olszyna nad rzeką Chociną między Katarzynkami a bagmen Czystym, na prawo od szosy, 4 May 1966, *S*. *Lisowski*, *F*. *Szafrański*, *K*. *Tobolewski*, POZG-B *61683* [36]; dolnośląskie voivodeship, Buczyna Jakubowska reserve, on the log in *Fraxino-Alnetum* forest, 13 Dec 2017, *G. J. Wolski*, LOD *14924* [Wolski15], Łęgi Źródliskowe koło Przemkowa reserve, on the log in *Tilio*-*Carpinetum* forest, 13 Dec 2017, *G. J. Wolski*, LOD *14926* [Wolski12]; łódzkie voivodeship, Zimna Woda reserve, on stump in *Ribeso nigri-Alnetum glutinosae* forest, 15 Dec 2017, *G. J. Wolski*, LOD *14933* [Wolski14], on the log in *Ribeso nigri-Alnetum*forest, 15 Dec 2017, *G. J. Wolski*, LOD *14934* [Wolski19]; warmińsko-mazurskie voivodeship, Kadyński Las reserve, on the stump in *Fraxino-Alnetum* forest, 13 Feb 2018, *G. J. Wolski*, LOD *14936* [Wolski17]. **RUSSIA**: Colchis, distr. Sochi, in vicinitate pagi Galycino, in valle fluminis Mzymta, alt. 300 m, 8 May 1985, *V*. *Vašák*, NY *3684217* [83]; Ingushetia, in valle fluminis Armchi, in vicinitate pagi Dzheirakh, alt. 1300 m, 5 Jul 1990, *V*. *Vašák*, NY *3103641* [81]; Khosta distr., reservatum naturale „Samshitovata rosca”, alt. 100–300 m, 16 Jun 1978, *V*. *Vašák*, NY *3103751* [88]; Lazarevskoie distr., in vicinitate pagi Golvinka, 3 km ab ostoi fluminis Shakhe, in faucibus rivuli, alt. 20–100 m, 25 Mar 1983, *V*. *Vašák*, NY *3103750* [87]; Moskva, Park Fili-Kuntzevo, old deciduous forest, in ravine at the slope of Moskva-River, steep bank of deep, wet and shaded ravine, alt. 120 m, 55°47ʹN, 37°24ʹE, 20 Jul 1990, *E*. *A*. *Ignatova* & *M*. *S*. *Ignatov*, NY *3103753* [101]; Moskva, Park Fili-Kuntzevo, old deciduous forest, in ravine at the slope of Moskva-River, steep bank of deep, wet and shaded ravine, alt. 120 m, 55°47ʹN, 37°24ʹE, 20 Jul 1990, *E*. *A*. *Ignatova* & *M*. *S*. *Ignatov*, NY *3684219* [102]; Karachaevo-Cherkessin Republic, Teberda Nature Reseve, Shumka Creek, near waterfall, *Abies*+*Fagus­* forest, on soil on steep slope, 43°24ʹ N, 41°44ʹ E, alt. 1500 m, 16 Sep. 2005, *M*. *Ignatov*, *E*. *Ignatova*, H*3226596* [130]. **SPAIN**: Cantabria, Celada Marlantes, monte Matanzas, 42°55ʹ53ʺN, 4°05ʹ56ʺW, alt. 1250 m, roca en hayedo, 22 Nov 2008, M .J. Cano, *4782*, NY 1273319 [77], BM1007993 [178]. **SWEDEN**: Blekinge, Förkärla par., Tromtö, På bergvägg, 21 Jun. 1888, *H*. *W*. *Arnell*, UPS B-*601048* [163], Karlskrona par., Vämmö, 23 Jun. 1888, *H*. *W*. *Arnell*, UPS B-*601051* [164]; Bohuslän, Marstrand, Marstrandsön, N om samhället, skuggig bergbrant, 27 Feb. 1983, *T*. *Hallingbäck*, S B*217137* [220]; Rödbo, Elleröd, 57,8513671° N, 11,9721981° E, På sluttande klippa i lövskog, 20 May 2016, *A*. *Stansvik*, S B*270146* [221]; Öland, Böda par., Böda kronopark i Bogateskogen, 26 Jul. 1867, *J*. *E*. *Zetterstedt*, UPS B-*601091* [166]; Skåne, Östra Vemmenhög par., Maltesholm, I barrskog, 28 Jun. 1944, *E*. *Nyholm*, UPS B-*585204* [167]; Västergötland, Berg, Nolberget, Nord-brant, 18 Mar. 1993, *T*. *Hallingbäck*, S B*217143* [219]. **SWITZERLAND**: Thurgau, an morschen Baumstrünken, Aug. 1862, *B*. *Schenk*, BM*1103876* [179]. **TURKEY**: Izmir Prov. Bozdağ south of the village, on bank of stream, alt. 1320 m, *E. Nyholm* & *A*. *C*. *Crundwell*, E*905524* [103]; S of the village Bozdağ, on stream bank, alt. 1300 m, 11 Apr 1971, *N*. *Ayedem*, *et al.*, NY *3103692* [104], in cave baside stream, 1971, *N*. *Ayedem*, *et al.*, H*3214342* [143], alt. 1300 m, on stream bank, 11 Apr. 1971, *N*. *Ayedem*, *et al.*, *923*/*71*, S B*68175* [200]; Trabzon Prov., ca 5 km S of Hamsiköy, Pontos Mt. S. facing slope bearing young trees of *Fagus*, *Cornus*, etc. in *Picea*-*Fagus* forest, 1959, *E*. *Hennipman*, *E*. *Nyholm*, S B*68173* [198]. **UNITED KINGDOM**: Jersey, Nez du Guet near Rozel, wooded slopes above sea, on shady mossy rocks, alt. 40 m, 2 May 2004, *D*. *G*. *Long*, *33156*, E*905425* [120], Greve de Lecq Valley, sandy stream bank, 30 Mar 1957, *A*. *C*. *Crundwell*, *253*, E*905424* [119]; Stirling, Cailness Glen. NN 34 06, 15 Apr 1967, *S*. *A*. *McGregor*, E*418247* [105]; Achill Island, Co. Mayo, Cornaclea Point, splashed by water, streamside on boulder-clay seacliff, 2 Aug. 1951, *S*. *Drake*-*Brockman*, BM*1103859* [205]; Smallbrook valley, Surrgy, *S*. *Edwards*, MANCH Kk *151* [203].

***Plagiothecium* *angusticellum***

Type: Poland. Łódzkie Voivodeship. Grądy nad Moszczenicą reserve. 51°55′N, 19°29′E, at the bottom part of *Carpinus betulus* in *Fraxino-Alnetum* forest, 11 Dec 2017, *G. J. Wolski* (holotype LOD*14927*, isotype LOD *14937*).

**CZECH REPUBLIC**: Southern Bohemia, Šumava Mts. NE slope of then Stožec mt. spruce forest, stones, alt. 800 m, Oct 1992, *J*. *Kučera*, PRC *198* [1]; Ceskomoravska vrchovina, Jihlava, 3 Aug 1974, *R*. *Doležal*, SNM *150* [3], Zdáeské vrchy, 0.8 km from Hluboká, 6 Jun 1978, *R*. *Doležal*, SNM *217* [6]; Doupovská pahorkatina, Oslovice, na břehu potoka Bublava, ca 150 m jižně silnice, alt. 360 m, 12 Oct 1989, *M*. *Vondráček*, PL *169/763* [10]; Doupovské vrchy, Kyselka, skalky v parku nad silnicí, alt. 460 m, 17 Jul 1989, *M*. *Vondráček*, PL *169/757* [8], Korunní Kyselka, údolí Korunního potoka, ca 500 m JV osady, alt. 430 m, 22 Jul 1989, *M*. *Vondráček*, PL *169/760* [7]; Morava, Brno, 1.3 km from Brno, 10 Aug 1979, *R*. *Doležal*, SNM *226* [2], Drahanská vrchovina, Brno, 19 Aug 1979, *R*. *Doležal*, SNM *226* [5]. **ESTONIA**: Saaremaam Abruka, 16 Jun 1935, *R*. *Tuomikuaski*, H*3113593* [56], H*3230093* [55]; Viljandimaa, Viljandi vald. Heimtali, salumersas, vahtra tüvealusel, 17 Jul 1996, *M*. *Leis*, TAA *5004954* [50]. **HUNGARY**: Nógrád, in rupibus umbrosis rivi Baciapatak, pr. Királyháza, mtes Börzsöny, 2 Jun 1957, *L*. *Vajda*, H*3113607* [28]. **LATVIA**: Limbaži district, Liepupes forestry, rich deciduous forest, on the base of black alder, 57°31ʹ49ʺN, 24°24ʹ56ʺE, 30 Sep 2004, *B*. *Bambe* SILAVA *24360* [69]. **LITHUANIA**: Jonavos r., apie 3 km I R nuo Žiemių, Žiemių miško P dalie, 14 kv., Drėgame uosyne, liepyne, 26 Jun 1999, *J*. *Ferencaitė*, BILAS *B7510* [63]; Kalvarijos sav., apie 6 km i ŠR nuo Mockų, Sŭsninkų miškas, 88 kv., 8 skl., Drėgname lapuočių miške, 27 Jul 2000, *V*. *Marozas*, BILAS *B8742* [58]. **POLAND**: Western Beskid Mts., Beskid Maly Mts., Kocierz Górny, buczyna, na glebie, na skarpie, 1994, *A*. *Stebel* & *M*. *Stebel*, SOSN *23652* [42]; Silesian Beskid, Ustroń Polana, *A*. *Stebel*, SOSN *34732* [41]; Central Poland, rezerwat Jodły Łaskie, gmina Sędziejowice, powiat łaski, łęg jesionowo-olszowy, 21 Jul 2003, *Sęczkowska*, LOD *12457* [45], rezerwat Grądy nad Moszczenicą, 19°30ʹN, 51°55ʹE, gmina Zgierz, powiat zgierski, województwo łódzkie, kora *Quercus robur*, Alno-Ulmion, 14 Jul 2010, *G*. *J*. *Wolski*, LOD *14553* [44], Radziwiłłów, gmina Puszcza Mariańska, ols, na glebie, 18 Aug 1968, *R*. *Olaczek*, LOD *7271* [46], uroczysko Walewice, gmina Sulmierzyce, źródlisko, 31 May 1985, *E*. *Filipiak*, LOD *7281* [43]; dolnośląskie voivodeship, Buczyna Jakubowska reserve, on the stump in *Fraxino-Alnetum* forest, 13 Dec 2017, *G. J. Wolski*, LOD *14923* [Wolski1], Łęgi Źródliskowe koło Przemkowa reserve, on the mineral soil in *Fraxino-Alnetum* forest, 13 Dec 2017, *G. J. Wolski*, LOD *14925* [Wolski23], łódzkie voivodeship, Grądy nad Moszcenicą reserve, on the bottom part of *Carpinus betulus* in *Fraxino-Alnetum* forest, 51°55ʹN, 19°29ʹE, 11 Dec 2017, *G. J. Wolski*, LOD *14927* [Wolski22], on the bottom part of *Alnus glutinosa* in *Fraxino-Alnetum* forest, 11 Dec 2017, *G. J. Wolski*, LOD *14928* [Wolski24], Wiąńczyń reserve, on mineral soil in *Luzulo pilosae-Fagetum* forest, 10 Oct 2016, *G. J. Wolski*, LOD *14929* [Wolski5], LOD *14930* [Wolski26], LOD *14931* [Wolski28], LOD *14932* [Wolski29]; Zimna Woda reserve, on the mineral soil in *Tilio*-*Carpinetum* forest, 12 Dec 2017, *G. J. Wolski*, LOD *14935* [Wolski25].
