# Supplementary material for: Resurrection of the Plagiothecium longisetum Lindb. and proposal of the new species—P. angusticellum
Source: PLoS One. 2020 Mar 11;15(3):e0230237. doi: 10.1371/journal.pone.0230237 (PMC7065767; doi:10.1371/journal.pone.0230237)
Supplement: S1 Table — A: partitions for ITS matrix (710 bp) analysis; B: ITS-rps4-rpl16 matrix (2068 bp) analysis. (DOCX) [file pone.0230237.s003.docx]

**S1 Table** **Summary of partitions for evolutionary model selection and phylogenetic interference using PartitionFinder2.** A: partitions for ITS matrix (710 bp) analysis; B: ITS-*rps4*-r*pl16* matrix (2068 bp) analysis.

| **A** | ITS1 | 5.8S gDNA | ITS2 |
| --- | --- | --- | --- |
| ML | GTR+G | K80 | K81UF+G |
| BI | GTR | K80 | GTR |

| **B** | ITS1 | 5.8S gDNA | ITS2 | *rps4* introne | *rps4* codone | *rpl16* introne | *rpl16* codone |
| --- | --- | --- | --- | --- | --- | --- | --- |
| ML | K80+G | JC | HKY+I | TIM+I+G | TIM | TIM+I+G | JC |
| BI | K80 | JC | HKY | GTR | GTR | GTR | JC |
