# Supplementary material for: Resurrection of the Plagiothecium longisetum Lindb. and proposal of the new species—P. angusticellum
Source: PLoS One. 2020 Mar 11;15(3):e0230237. doi: 10.1371/journal.pone.0230237 (PMC7065767; doi:10.1371/journal.pone.0230237)
Supplement: S2 Table — LC1, LC2, LC3, WC1, WC2, WC3 –explanation in Table 1; N–number of observations, x¯ –mean, Me–median, Min–minimum, Max–maximum, Q1 –first quartile, Q3 –third quartile. Data (x¯, Me, Min, Max) are given in μm. (DOC) [file pone.0230237.s004.doc]

**S2 Table Descriptive statistics of the examined specimens of *Plagiothecium nemorale sensu lato*.**

| Feature | N | x̄ | Me | Min | Max | Q1 | Q3 |
| --- | --- | --- | --- | --- | --- | --- | --- |
| LC1 | 240 | 91.97 | 88.85 | 57.90 | 158.10 | 76.95 | 103.25 |
| WC1 | 240 | 22.20 | 21.90 | 11.60 | 33 | 18.30 | 25.90 |
| LC2 | 240 | 113.54 | 116.65 | 67.60 | 150.30 | 93.95 | 130 |
| WC2 | 240 | 23.10 | 22.70 | 12.10 | 34.10 | 19.30 | 26.35 |
| LC3 | 240 | 138.88 | 138.40 | 77.70 | 223.10 | 120.70 | 156.55 |
| WC3 | 240 | 26.12 | 25.25 | 15.90 | 43.30 | 21.35 | 30.10 |

LC1, LC2, LC3, WC1, WC2, WC3 – explanation in Table 1; N – number of observations, x̄ – mean, Me – median, Min – minimum, Max – maximum, Q1 – first quartile, Q3 – third quartile. Data (x̄, Me, Min, Max) are given in µm.
