# Supplementary material for: Resurrection of the Plagiothecium longisetum Lindb. and proposal of the new species—P. angusticellum
Source: PLoS One. 2020 Mar 11;15(3):e0230237. doi: 10.1371/journal.pone.0230237 (PMC7065767; doi:10.1371/journal.pone.0230237)
Supplement: S3 Table — LC1, LC2, LC3, WC1, WC2, WC3 –explanation in Table 1; N–number of observations, x¯ –mean, Me–median, Min–minimum, Max–maximum, Q1 –first quartile, Q3 –third quartile. Data (x¯, Me, Min, Max) are given in μm. (DOC) [file pone.0230237.s005.doc]

**S3 Table Descriptive statistics of individual characteristics of the examined species**.

| Feature | Species | N | x̄ | Me | Min | Max | Q1 | Q3 | SD |
| --- | --- | --- | --- | --- | --- | --- | --- | --- | --- |
| LC1 | *P*. *nemorale*  *sensu stricto* | 115 | 78.17 | 77.80 | 57.90 | 124.30 | 70.20 | 84.70 | 11.30 |
| WC1 |  | 115 | 21.49 | 20.90 | 11.60 | 33.00 | 17.90 | 25.10 | 4.75 |
| LC2 |  | 115 | 96.76 | 93.30 | 67.60 | 131.80 | 87.80 | 106.30 | 13.21 |
| WC2 |  | 115 | 22.20 | 21.40 | 12.10 | 32.10 | 19.50 | 25.20 | 4.02 |
| LC3 |  | 115 | 121.63 | 120.40 | 77.70 | 195.90 | 104.00 | 138.20 | 21.92 |
| WC3 |  | 115 | 25.02 | 23.80 | 15.90 | 43.30 | 20.70 | 28.60 | 5.80 |
| LC1 | *P*. *longisetum* | 125 | 104.67 | 102.50 | 68.50 | 158.10 | 93.30 | 114.20 | 16.26 |
| WC1 |  | 125 | 22.86 | 23.30 | 13.20 | 32.30 | 19.00 | 26.40 | 4.67 |
| LC2 |  | 125 | 128.97 | 129.40 | 94.60 | 150.30 | 122.60 | 136.20 | 9.53 |
| WC2 |  | 125 | 23.93 | 24.00 | 15.10 | 34.10 | 19.20 | 27.40 | 4.99 |
| LC3 |  | 125 | 154.74 | 151.30 | 96.10 | 223.10 | 137.20 | 169.30 | 20.70 |
| WC3 |  | 125 | 27.14 | 27.30 | 16.40 | 40.20 | 21.90 | 31.90 | 5.71 |

LC1, LC2, LC3, WC1, WC2, WC3 – explanation in Table 1; N – number of observations, x̄ – mean, Me – median, Min – minimum, Max – maximum, Q1 – first quartile, Q3 – third quartile, SD – standard deviation. Data (x̄, Me, Min, Max) are given in µm.
