# Supplementary material for: Resurrection of the Plagiothecium longisetum Lindb. and proposal of the new species—P. angusticellum
Source: PLoS One. 2020 Mar 11;15(3):e0230237. doi: 10.1371/journal.pone.0230237 (PMC7065767; doi:10.1371/journal.pone.0230237)
Supplement: S4 Table — LC1, LC2, LC3, WC1, WC2, WC3 –explanation in Table 1; N–number of observations, x¯ –mean, Me–median, Min–minimum, Max–maximum, Q1 –first quartile, Q3 –third quartile. Data (x¯, Me, Min, Max) are given in μm. (DOC) [file pone.0230237.s006.doc]

**S4 Table** Descriptive statistics of individual features of the studied taxa.

| Feature | Taxon | N | x̄ | Me | Min | Max | Q1 | Q2 | SD |
| --- | --- | --- | --- | --- | --- | --- | --- | --- | --- |
| LC1 | *P*. *angusticellum* | 30 | 103.79 | 104.85 | 81.70 | 120.40 | 97.70 | 111.90 | 10.32 |
| WC1 |  | 30 | 16.76 | 17.00 | 13.20 | 19.50 | 16.20 | 17.60 | 1.42 |
| LC2 |  | 30 | 125.98 | 124.80 | 113.00 | 143.30 | 121.60 | 129.40 | 6.72 |
| WC2 |  | 30 | 17.43 | 17.45 | 15.10 | 19.30 | 16.80 | 18.20 | 1.21 |
| LC3 |  | 30 | 140.88 | 137.85 | 123.10 | 172.00 | 134.60 | 147.20 | 11.00 |
| WC3 |  | 30 | 20.31 | 20.55 | 16.40 | 24.60 | 19.00 | 21.50 | 1.81 |
| LC1 | *P*. *longisetum* | 95 | 104.95 | 102.50 | 68.50 | 158.10 | 92.30 | 116.50 | 17.77 |
| WC1 |  | 95 | 24.79 | 24.60 | 17.00 | 32.30 | 22.10 | 27.80 | 3.54 |
| LC2 |  | 95 | 129.92 | 132.00 | 94.60 | 150.30 | 123.20 | 137.40 | 10.11 |
| WC2 |  | 95 | 25.98 | 25.80 | 17.00 | 34.10 | 23.50 | 29.00 | 3.83 |
| LC3 |  | 95 | 159.12 | 157.20 | 96.10 | 223.10 | 143.30 | 175.60 | 21.14 |
| WC3 |  | 95 | 29.30 | 28.80 | 19.90 | 40.20 | 25.60 | 32.70 | 4.74 |

LC1, LC2, LC3, WC1, WC2, WC3 – explanation in Table 1; N – number of observations, x̄ – mean, Me – median, Min – minimum, Max – maximum, Q1 – first quartile, Q3 – third quartile, SD – standard deviation. Data (x̄, Me, Min, Max) are given in µm.
