# Supplementary material for: Resurrection of the Plagiothecium longisetum Lindb. and proposal of the new species—P. angusticellum
Source: PLoS One. 2020 Mar 11;15(3):e0230237. doi: 10.1371/journal.pone.0230237 (PMC7065767; doi:10.1371/journal.pone.0230237)
Supplement: S5 Table — (DOC) [file pone.0230237.s007.doc]

**S5 Table The results of the Student's t-test analysis together with the effect measure (Cohen's d) for the *P*. *longisetum* and *P*. *angusticellum*.**

| Feature | t | df | p (Cochran-Cox) | Cohen's d | Effect size |
| --- | --- | --- | --- | --- | --- |
| LC1 | -0.441 | 85.589 | 0.66 | 0.092 | no effect |
| WC1 | -17.986 | 116.3 | <0.001 | 3.767 | large |
| LC2 | -2.451 | 73.639 | 0.017 | 0.513 | intermediate |
| WC2 | -18.979 | 122.995 | <0.001 | 3.975 | large |
| LC3 | -6.169 | 95.9 | <0.001 | 1.292 | large |
| WC3 | -15.293 | 118.769 | <0.001 | 3.203 | large |
