# Supplementary material for: Resurrection of the Plagiothecium longisetum Lindb. and proposal of the new species—P. angusticellum
Source: PLoS One. 2020 Mar 11;15(3):e0230237. doi: 10.1371/journal.pone.0230237 (PMC7065767; doi:10.1371/journal.pone.0230237)
Supplement: S9 Table — Highlighted the distance between P. nemorale, P. longisetum and P. angusticellum specimens (asterisk indicate specimens described as P. angusticellum). (DOCX) [file pone.0230237.s011.docx]

**S9Table Genetic distance between *Plagiothecium* taxa based on *rpl16* part of the matrix.** Highlighted the distance between *P*. *nemorale*, *P*. *longisetum* and *P*. *angusticellum* specimens (asterisk indicate specimens described as *P*. *angusticellum*).
