# Supplementary material for: Resurrection of the Plagiothecium longisetum Lindb. and proposal of the new species—P. angusticellum
Source: PLoS One. 2020 Mar 11;15(3):e0230237. doi: 10.1371/journal.pone.0230237 (PMC7065767; doi:10.1371/journal.pone.0230237)

**S1 Fig Distributions of variables for cell length (A) and width (B) *Plagiothecium nemorale sensu lato*.** The values of the x axis are given in µm.


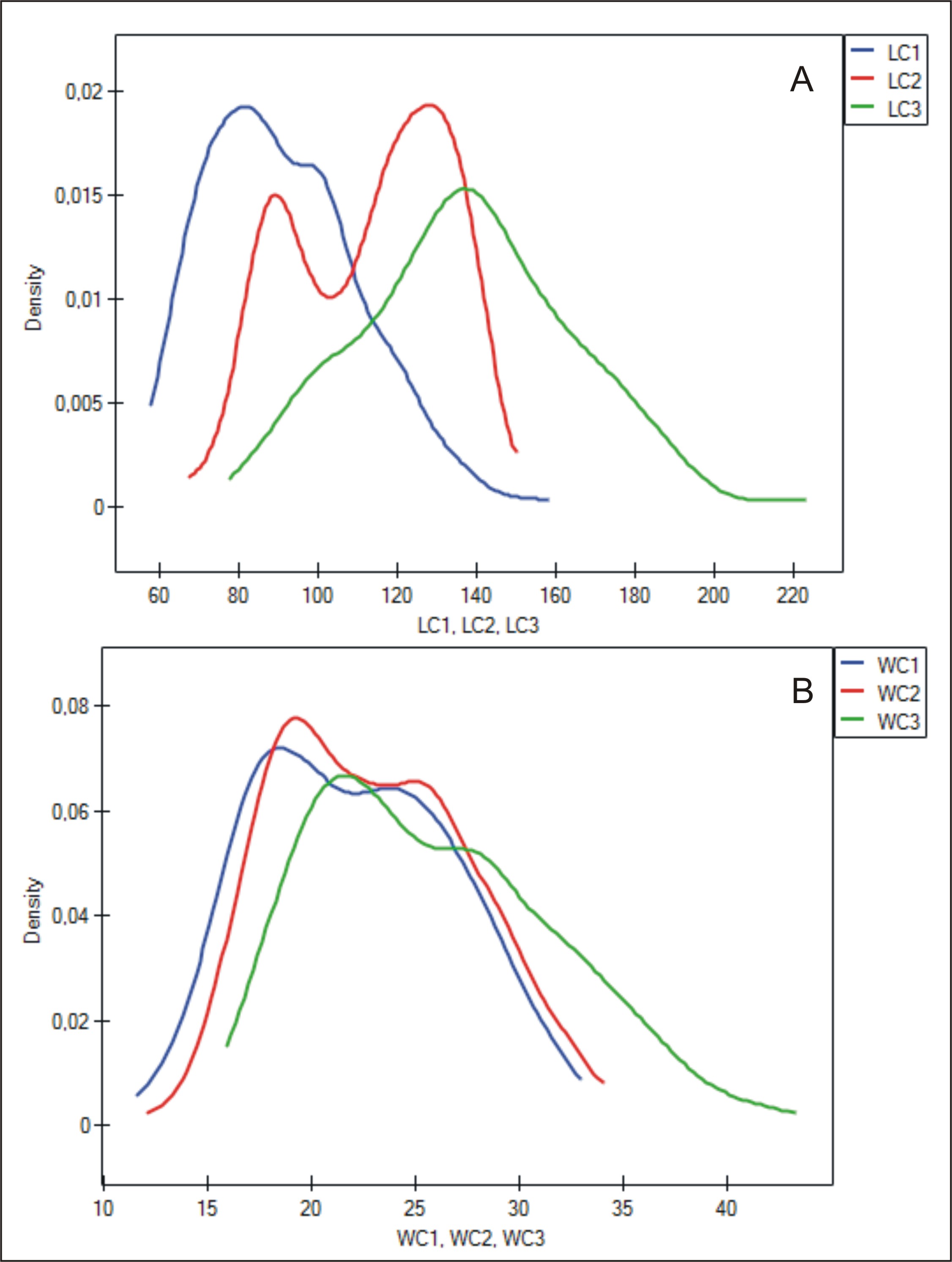

Supplement: S1 Fig — The values of the x axis are given in μm. (DOC) [file pone.0230237.s012.doc]
