# Supplementary material for: Resurrection of the Plagiothecium longisetum Lindb. and proposal of the new species—P. angusticellum
Source: PLoS One. 2020 Mar 11;15(3):e0230237. doi: 10.1371/journal.pone.0230237 (PMC7065767; doi:10.1371/journal.pone.0230237)

**S2 Fig Distributions of variables for individual species.** Red line – *P*. *nemorale* *sensu stricto*, green line – *P*. *longisetum*. The values of the x axis are given in µm.


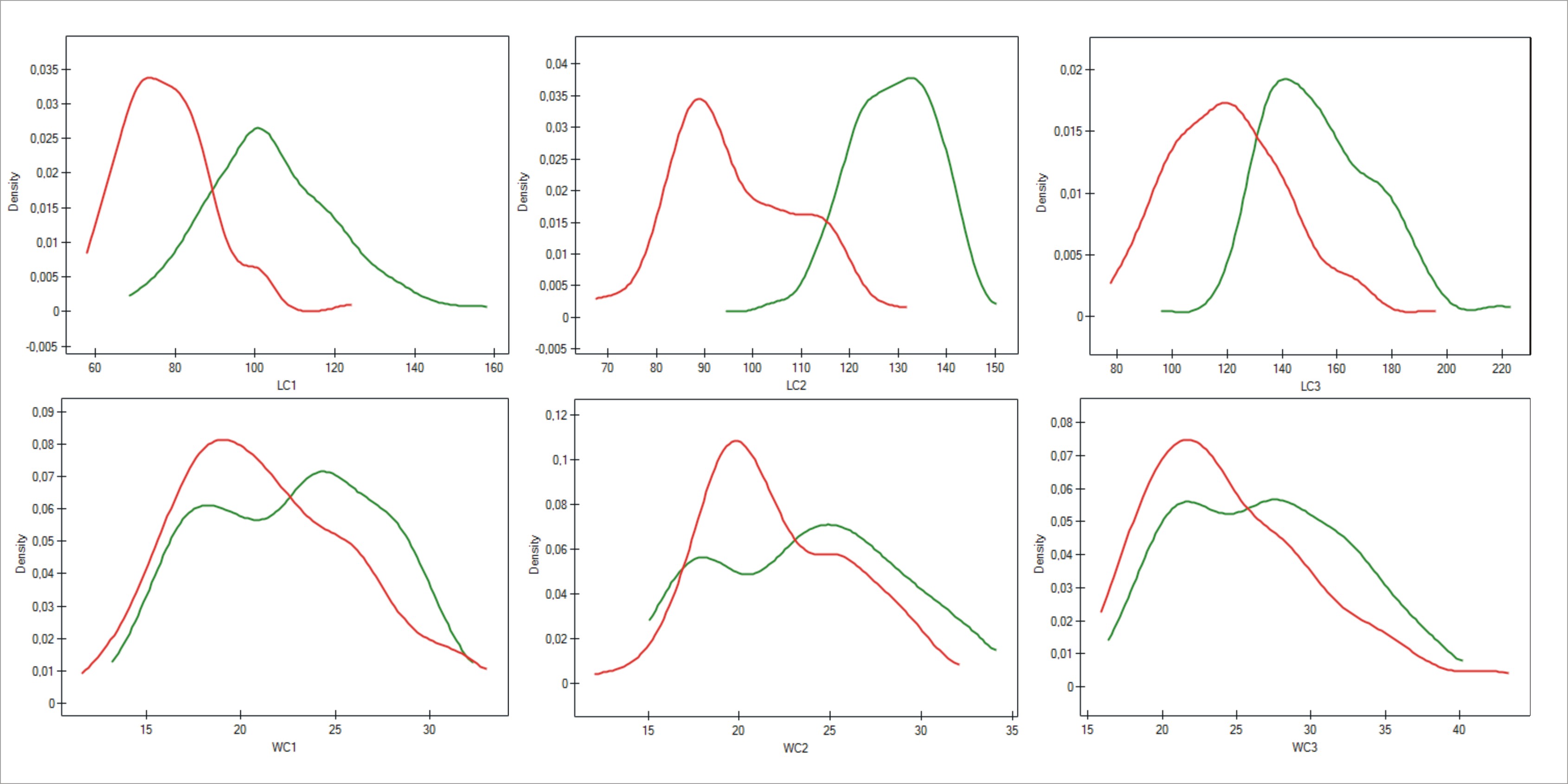

Supplement: S2 Fig — Red line–P. nemorale sensu stricto, green line–P. longisetum. The values of the x axis are given in μm. (DOC) [file pone.0230237.s013.doc]
